# Single dose VSV-based vaccine protects mice against lethal heterologous Crimean-Congo hemorrhagic fever virus challenge

**DOI:** 10.1038/s41541-025-01164-3

**Published:** 2025-05-30

**Authors:** Thomas Tipih, Shanna S. Leventhal, Kimberly Meade-White, Matthew Lewis, Trenton Bushmaker, Carl Shaia, Andrea Marzi, Heinz Feldmann, David W. Hawman

**Affiliations:** 1 Laboratory of Virology, Division of Intramural Research, National Institute of Allergy and Infectious Diseases, National Institutes of Health, Hamilton, MT USA; 2 Rocky Mountain Veterinary Branch, Division of Intramural Research, National Institute of Allergy and Infectious Diseases, National Institutes of Health, Hamilton, MT USA; 3 Present Address: HDT Bio, Seattle, Washington, USA

**Keywords:** Biotechnology, Vaccines, Virology

## Abstract

Crimean-Congo hemorrhagic fever virus (CCHFV) causes a severe, sometimes fatal hemorrhagic fever (CCHF) in humans. Currently, there are no approved therapies against CCHF. In this study we used the recombinant vesicular stomatitis virus (VSV) platform to generate live-attenuated recombinant CCHF vaccine candidates expressing the CCHFV nucleoprotein (NP) and glycoprotein precursor (GPC). As one approach, we utilized the established VSV expressing the full-length Ebola virus glycoprotein (VSV-EBOV) or a truncated version of the EBOV glycoprotein and added the CCHFV-NP (VSV-CCHFnp1 or VSV-CCHFnp2, respectively). Additionally, we prepared a vaccine candidate, VSV-CCHFgpc, in which the VSV glycoprotein was replaced with the CCHFV-GPC. Vaccine constructs induced CCHFV-specific IgG antibodies comprising largely IgG2c subclass. Only, the VSV-CCHFgpc vaccine candidate induced significant T cell immune responses directed against epitopes in the CCHFV-NSm and Gc proteins. Efficacy of the vaccine candidates was evaluated using a prime-only approach in a transiently immune-suppressed mouse model. Animals vaccinated with VSV-CCHFnp2 succumbed to lethal CCHFV challenge, while the VSV-CCHFgpc vaccine candidate afforded partial protection. In contrast, vaccination with VSV-CCHFnp1 uniformly protected animals against death. Our results demonstrate the promise of VSV-CCHFnp1 as a vaccine candidate for CCHFV and warrant continued development.

## Introduction

Crimean-Congo hemorrhagic fever virus (CCHFV) belongs to the *Orthonairovirus* genus, *Nairoviridae* family and possesses a tri-segmented single stranded RNA genome of negative polarity. The three segments are designated small (S) encoding the nucleoprotein (NP), medium (M) encoding the glycoprotein precursor (GPC), and large (L) encoding RNA-dependent RNA-polymerase^1^. GPC undergoes serial cleavage by host proteases into structural proteins (Gn and Gc), non-structural M protein (NS_M_)^2^, secreted non-structural proteins (GP160, GP85, GP38)^3,4^, and uncharacterized proteins (GPmuc and ProGc)^5^. CCHFV is the etiological agent of Crimean-Congo hemorrhagic fever (CCHF), a tickborne viral zoonosis. The disease is widespread in Africa, Asia, the Middle East, and Eastern Europe^6^, overlapping with the described distribution of ticks of the *Hyalomma* genus. CCHFV circulates in nature in a life cycle involving ticks and vertebrate animals that serve as amplifying hosts. *Hyalomma* ticks are regarded as the principal viral vector and reservoir^7,8^. Studies report CCHFV antibodies in wild and domestic animals^9^ without apparent disease, underscoring the role of animals in CCHFV epidemiology. Human infections are a result of bites from infected ticks, exposure to blood or tissue of infected livestock or healthcare associated. In humans, CCHFV often results in asymptomatic or subclinical infections but may result in CCHF with varying case fatality rates, typically in the range of 5–30%^10^.

Currently, there are no internationally recognized specific therapies or vaccines against CCHF. Therefore, there is a need for a vaccine to prevent disease in humans. Bulgaria uses an inactivated vaccine prepared from the brain tissue of CCHFV-infected newborn mice^11^. Vaccine efficacy, however, is yet to be established in controlled clinical trials. Furthermore, safety concerns and production methods will limit the utility of this vaccine elsewhere. Several other vaccine candidates are in preclinical development^6,12,13^. Even though vaccine-mediated immune correlates of protection are yet to be described, efforts on CCHF vaccine development have focused on the CCHFV glycoproteins and NP. The surface glycoproteins elicit neutralizing antibodies, but studies in animal models suggest that neutralizing antibodies are not required for protection against CCHFV infection^14,15^. The CCHFV-M segment exhibits the greatest nucleotide diversity of the three segments^10^ complicating efforts to develop a broadly protective CCHF vaccine. The NP is produced in large amounts during infection and is highly immunogenic^16^. Despite inducing non-neutralizing antibodies only, vaccine studies targeting the NP have been reported protective against CCHFV in animal models^17–19^. The NP amino acid sequence shows the least variation among isolates^20^ thus, an NP-based vaccine is hypothesized to offer broad protection against diverse CCHFV strains.

Recombinant vesicular stomatitis virus (VSV) expressing foreign glycoproteins (GP) have shown promise as experimental vaccines for several viral pathogens^21–24^. Advantages of VSV as a vaccine platform include its ability to grow to high titers, capability to induce innate and adaptive immune responses as well as the demonstrated safety profile^25–27^. Accordingly, the VSV-EBOV is approved for vaccination against Ebola virus (EBOV) (Ervebo, Merck)^28^. In this study, we used the VSV platform to generate live-attenuated recombinant CCHFV vaccine candidates. As vaccine antigens, we selected the CCHFV-NP and GPC that are known to confer protective immune responses in CCHF animal models studying vaccine efficacy^6,17^. The protective efficacy of the vaccine candidates was evaluated in mice vaccinated with a single dose of the recombinant VSV candidates and challenged with either the homologous CCHFV strain Hoti or a heterologous CCHFV strain UG3010. The VSV-CCHFgpc vaccine candidate induced neutralizing antibody responses and cellular immunity and was efficacious against homologous challenge, but could only afford partial protection against heterologous challenge. The VSV-CCHFnp1 vaccine elicited robust non-neutralizing IgG antibody responses and uniformly protected mice against heterologous lethal CCHFV challenge. Our results support further development of VSV-CCHFnp1 as a vaccine candidate against CCHF.

## Results

### Preparation and characterization of recombinant VSV vaccine candidates

**Fig. 1:**
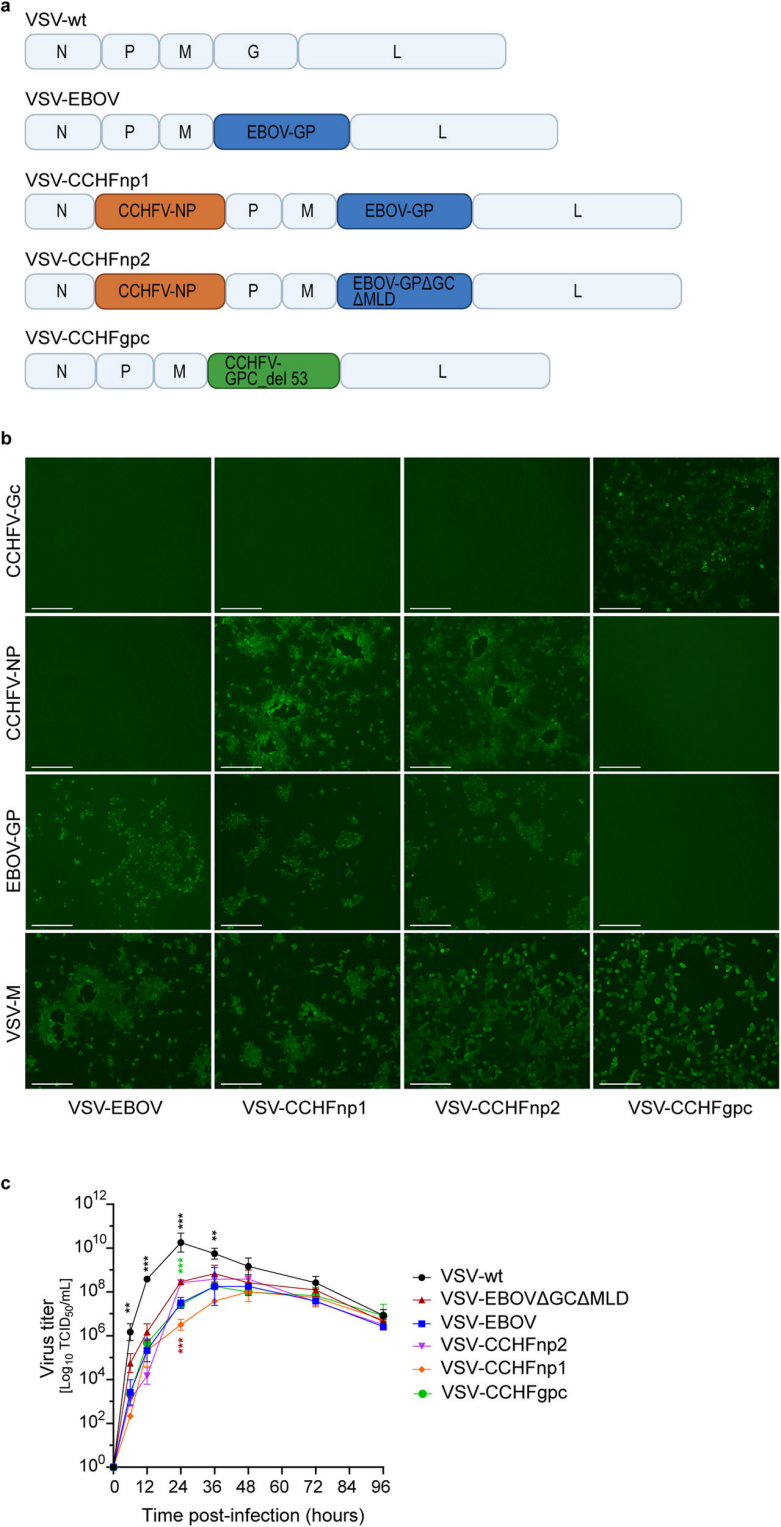
VSV vector design and characterization. **a** Schematic representation depicting the genomic organization of the VSV vectors. N nucleoprotein, P phosphoprotein, M matrix protein, G glycoprotein, L polymerase, EBOV-GP Ebola virus full-length glycoprotein, EBOV GP∆GC∆MLD Ebola virus glycoprotein lacking the glycan cap and mucin-like domain, CCHFV-NP CCHFV full-length nucleocapsid protein, CCHFV-GPC_del53 CCHFV glycoprotein precursor lacking 53 amino acid sequences of the carboxy terminal (see also Supplementary Fig. 1A). **b** Intracellular protein expression. Vero E6 cells were infected with each virus at an MOI of 0.01. CCHFV-NP, Gc, and EBOV-GP expression was detected by immunofluorescence following permeabilization. VSV-M served as a control (magnification, 10x). **c** In vitro growth kinetics of the VSV vectors. Vero E6 cells were infected with VSV vectors at an MOI of 0.01. Cell culture supernatants were collected at the indicated time points. The infectious virus was titrated using a TCID_50_ assay. One representative experiment in triplicate is shown. Statistical significance was analyzed using one-way ANOVA with Tukey’s multiple comparisons test in Prism 10 (GraphPad), and results are indicated as *p < 0.05, **p < 0.01, and ***p < 0.001. Comparisons with *p* values >0.05 were not displayed. Data shown as geometric mean plus standard deviation. Graphical illustrations were prepared with Adobe Illustrator version 28.7 (public domain).

To prepare constructs expressing the CCHFV-NP, we utilized the established recombinant VSV platform expressing either the full-length Ebola virus glycoprotein (VSV-EBOV)^29–31^ or the recombinant VSV-EBOVΔGCΔMLD^31^ that harbors deletions of the glycan cap (GC) and mucin-like domain (MLD) which carry important immunodominant regions of the EBOV-GP^32–34^ to prepare constructs expressing the CCHFV-NP. The latter approach was chosen to skew immune responses toward the CCHFV-NP. The gene coding the full-length open reading frame of the CCHFV-NP from strain Hoti was ligated into either the VSV-EBOV (VSV-CCHFnp1) or the recombinant VSV-EBOVΔGCΔMLD plasmid (VSV-CCHFnp2) (Fig. 1a). Sequencing confirmed that no mutations were observed in VSV-CCHFnp1 and VSV-CCHFnp2. Additionally, we prepared the VSV-CCHFgpc vaccine candidate by replacing the gene encoding the VSV glycoprotein with the CCHFV strain Hoti GPC gene (Fig. 1a). The CCHFV-GPC gene used possesses a deletion of 53 amino acids off the carboxy terminal region of the Gc previously shown to promote rescue of VSV-CCHFgpc but retaining the Gc transmembrane domain^35^, designated CCHFV-GPC_del53. The VSV-CCHFgpc vaccine candidate grew poorly and was therefore passaged four times in Vero E6 cells to enhance viral titers. Deep sequencing of the passaged VSV-CCHFgpc revealed one synonymous mutation at nucleotide position 1122 (S374S) and two non-synonymous mutations (R520K & N759T) in the open reading frame of the CCHFV-GPC (Supplementary Fig. 1a). In vitro expression of CCHFV-NP, CCHFV-GPC, EBOV-GP, and VSV-M was confirmed by immunofluorescence assay (Fig. 1b) and western blot (Supplementary Figs. 1b, c, 2) on Vero E6 cells infected with the different recombinant VSV vaccine candidates.

To demonstrate in vitro attenuation of the recombinant VSV vaccine candidates, we compared virus growth kinetics on Vero E6 cells at various time points up to 96 h post infection (PI). Infectious virus from the cell culture supernatant was titrated using a TCID_50_ assay. The VSV wildtype (VSV-wt) grew rapidly and achieved significantly higher viral titers between 6–36 h PI compared to the recombinant VSV vaccine candidates (Fig. 1c). As expected, VSV-CCHFnp1 and VSV-CCHFnp2 displayed slower growth kinetics compared to the VSV-wt. In line with greater attenuation, the recombinant VSV vaccine candidate encoding the full-length EBOV-GP gene attained significantly lower viral titers compared to the recombinant VSV vaccine candidate encoding the truncated EBOV-GP gene at 24 h PI. The VSV-CCHFgpc vaccine candidate achieved peak viral titers at 36 h PI, as has been reported before^36^.

### VSV-CCHFgpc vaccination induces significant B cell and T cell responses

**Fig. 2:**
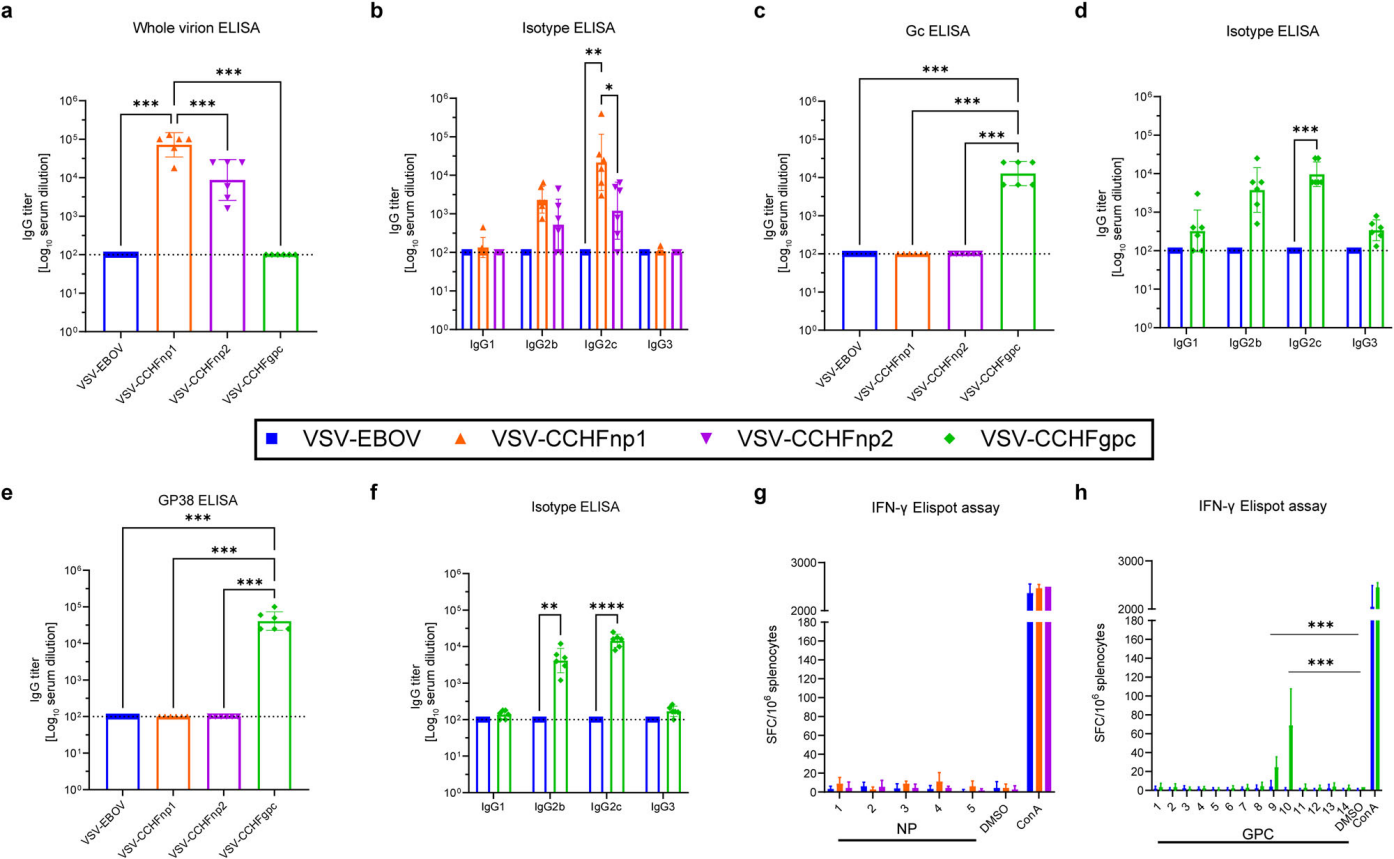
VSV-CCHFgpc vaccination induces significant B cell and T cell responses. Six-week-old C57BL/6J mice were vaccinated intraperitoneally with 1 × 10^4^ PFU VSV-CCHFnp1 or VSV-CCHFnp2 or VSV-CCHFgpc or VSV-EBOV on day -28. On day 0, groups of six mice were euthanized and blood and spleen samples were collected for evaluation of immune responses. Whole virion ELISA (**a**) or specific isotypes (**b**) was used to detect IgG responses elicited by the CCHFV-NP vaccines. **c** CCHFV-Gc specific IgG antibodies were evaluated by recombinant ELISA or **d** specific isotypes. CCHFV-GP38-specific IgG antibodies were evaluated by recombinant ELISA (**e**) or specific isotypes (**f**). Dashed line represents the cut-off for seropositivity, which was set at 3 standard deviations above the mean absorbance of wells that received no serum. **g**, **h** CCHFV-specific T cell responses were measured using IFN-γ ELISpot. The number of spot-forming cells against individual CCHFV-NP (**g**) or GPC peptide pools (**h**), the mitogen concanavalin A or DMSO vehicle alone are shown. Statistical significance was calculated using one-way ANOVA with Tukey’s multiple comparisons test (**a**, **c**, **e**) or two-way ANOVA with Sidak’s multiple comparisons test (**b**, **d**, **f**, **g**, **h**) and results are indicated as *p < 0.05, **p < 0.01, and ***p < 0.001. Comparisons with *p* values >0.05 were not displayed. Data shown as geometric mean plus standard deviation.

To determine whether the recombinant VSV vaccine candidates were immunogenic, wild-type C57BL/6J mice were vaccinated intraperitoneally (IP) with 10^4^ PFU following a prime-only approach (Supplementary Fig. 3). Serum samples from a subset of vaccinated mice were evaluated for the presence of CCHFV-specific antibodies on day 28 post vaccination (Fig. 2a–f). CCHFV-NP specific IgG antibodies were detected in all VSV-CCHFnp1 and VSV-CCHFnp2 vaccinated animals, with significantly higher responses in the former (Fig. 2a). Anti-CCHFV IgG NP antibodies were mostly from the IgG2c subclass (Fig. 2b). The VSV-CCHFgpc vaccine candidate induced CCHFV Gc- and GP38-specific IgG antibodies, predominantly IgG2c subclasses in immunized animals (Fig. 2c–f), while Gn specific IgG antibodies were low or undetectable (Supplementary Fig. 4a). Taken together, IgG subclass profiling suggests induction of a predominant T helper 1 response. As expected, neither CCHFV-NP nor Gc- nor Gn- nor GP38-specific antibodies were measured in sera from mice vaccinated with the VSV-EBOV vaccine.

To examine the ability of the antibody response to neutralize the homologous CCHFV strain Hoti and heterologous CCHFV strain UG3010, we tested mice sera using the microneutralization assay. Mice vaccinated with VSV-CCHFgpc but not VSV-CCHFnp1 or VSV-CCHFnp2 generated low levels of neutralizing antibody response against both CCHFV strain Hoti (Supplementary Fig. 4b) or the CCHFV strain UG3010 (Supplementary Fig. 4c), confirming that VSV-CCHFgpc elicited antibodies capable of neutralizing both homologous and heterologous CCHFV strains. We also analyzed EBOV-GP-specific IgG antibodies in vaccinated mice (Supplementary Fig. 4d). Vaccinating animals with the VSV-CCHFnp1 vaccine resulted in higher, but not statistically different, EBOV-GP-specific antibody titers compared to VSV-CCHFnp2.

To investigate CCHFV-specific T cell responses following immunization with the recombinant VSV vaccine candidates, splenocytes were harvested from vaccinated mice euthanized on day 28 post-vaccination and stimulated in vitro with peptide pools spanning CCHFV Hoti NP or GPC. IFN-γ secretion by stimulated splenocytes were evaluated as an index of T cell responses. No significant CCHFV-specific T cell response was measured in mice vaccinated with the VSV-CCHFnp1 or the VSV-CCHFnp2 compared to the VSV-EBOV immunized group (Fig. 2g). In contrast, immunizations with the VSV-CCHFgpc candidate vaccine induced a significant IFN-γ T cell response against CCHFV-GPC peptide pools 9 and 10 (Fig. 2h), which span the C-terminus of the NSm protein and the N-terminus of the Gc protein. Cumulatively, our immunological analyses of VSV-based CCHFV-NP and GPC vaccine candidates show that VSV-CCHFnp1 vaccination primarily elicited a robust non-neutralizing antibody response while VSV-CCHFgpc elicited a modest neutralizing antibody response and cellular immunity against epitopes in the CCHFV-NSm and Gc proteins.

### VSV-CCHFnp1 vaccine protects mice from death against heterologous CCHFV challenge

**Fig. 3:**
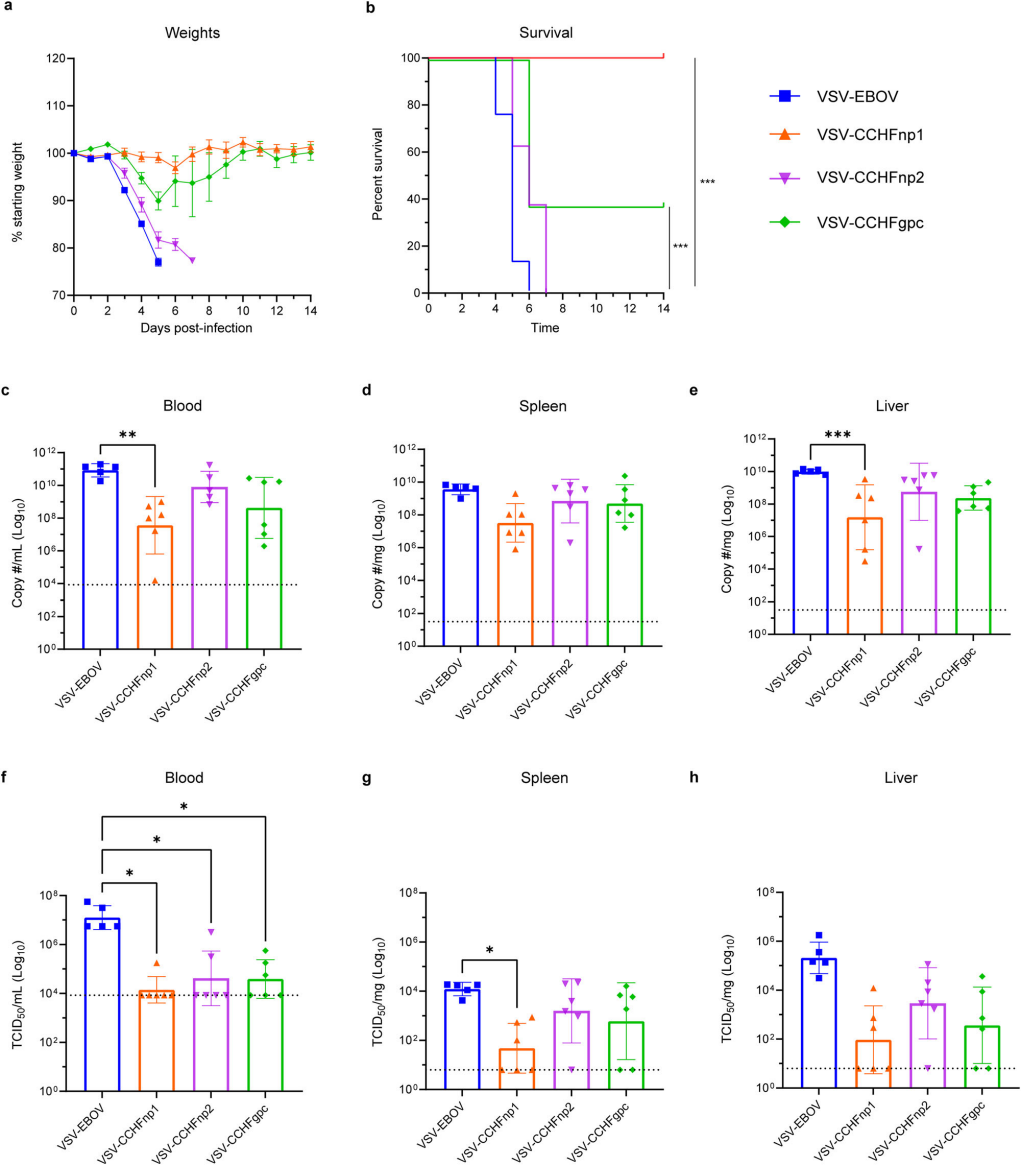
VSV-CCHFnp1 vaccine protects mice from death against heterologous CCHFV challenge. Six-week-old C57BL/6J mice were vaccinated intraperitoneally (IP) with 1 × 10^4^ PFU VSV-CCHFnp1 or VSV-CCHFnp2 or VSV-CCHFgpc or VSV-EBOV on day -28 relative to challenge. On day 0, mice were treated IP with MAR1-5A3 to block type I IFN receptor signaling and challenged IP with 100 TCID_50_ CCHFV strain UG3010. Mice were weighed daily (**a**), and monitored for survival (**b**). *N* = 8 mice per group. **c**–**e** Viral RNA in indicated tissues at day 5 post challenge was quantified by RT-qPCR. **f**–**h** Replicating virus in indicated tissues at day 5 post challenge was quantified by a TCID_50_ assay. *N* = 6 mice per group. One animal in the VSV-EBOV group succumbed during viral challenge at day 0 (cause unknown), leaving five animals in the group. The dashed line indicates the limit of detection. Statistical comparisons were calculated using log-rank test with Bonferroni’s correction for multiple comparisons test (**b**) one-way ANOVA with Tukey’s multiple comparisons test (**c**–**h**) and results are indicated as *p < 0.05, **p < 0.01, ***p < 0.001, and ****p < 0.0001. Comparisons with *p* values >0.05 were not displayed. Data shown as geometric mean plus standard deviation.

Next, we evaluated the protective efficacy of the VSV-CCHF vaccine candidates against a heterologous CCHFV strain UG3010 challenge. CCHFV strain UG3010 differs in amino acid sequence from the vaccine-encoded CCHFV Hoti GPC by >25% and from the NP by 4.5%^17^. Animals were immunized (IP) with 10^4^ PFU of VSV-CCHFnp1 or VSV-CCHFnp2 or VSV-CCHFgpc vaccine candidates. Control group animals received 10^4^ PFU of the VSV-EBOV vaccine. Twenty-eight days after vaccinations, animals were treated with MAR1-5A3, a type I IFN receptor blockade antibody to render the mice susceptible to CCHFV infection^37^, and were subsequently challenged (IP) with a lethal dose of the CCHFV strain UG3010 (100 TCID_50_) as established previuosly^17^. One animal in the VSV-EBOV group succumbed during viral challenge at day 0 (cause unknown), leaving five animals in the group. Following viral challenge, mice were monitored daily for the development of disease symptoms. As expected, mice in the control group lost weight beginning day 3 PI (Fig. 3a) and all succumbed by day 6 PI (Fig. 3b). Surprisingly, mice vaccinated with VSV-CCHFnp2 progressively lost weight beginning day 3 PI and (Fig. 3a) all succumbed by day 7 PI (Fig. 3b). Disease was delayed in VSV-CCHFgpc vaccinated animals compared to the VSV-EBOV and VSV-CCHFnp2 groups with animals losing weight starting day 4 PI and 5 out of 8 animals succumbed to challenge by day 6 PI (Fig. 3a, b). In contrast, vaccination with VSV-CCHFnp1 prevented weight loss and uniformly protected all the animals against lethal disease (Fig. 3a, b).

We collected blood, liver, and spleen samples from challenged animals on day 5 PI and evaluated viral loads as measured by RT-qPCR and titration assays. Viral RNA was detected in control and vaccine groups, including the VSV-CCHFnp1 group, suggesting CCHFV replication (Fig. 3c–e). However, statistically reduced viral RNA copy numbers were only seen in the VSV-CCHFnp1 group compared to the VSV-EBOV group. Similarly, infectious virus was detected in all vaccinated animals. Even though animals had significantly reduced infectious CCHFV in the blood, infectious titers in the spleen were only significantly different for the VSV-CCHFnp1 but not the VSV-CCHFnp2 and VSV-CCHFgpc mice compared to VSV-EBOV vaccinated mice (Fig. 3F–H). Taken together, these results show that VSV-CCHFnp1 conferred significant protection against clinical disease that correlated with significantly reduced viral RNA and infectious virus in key tissues.

**Fig. 4:**
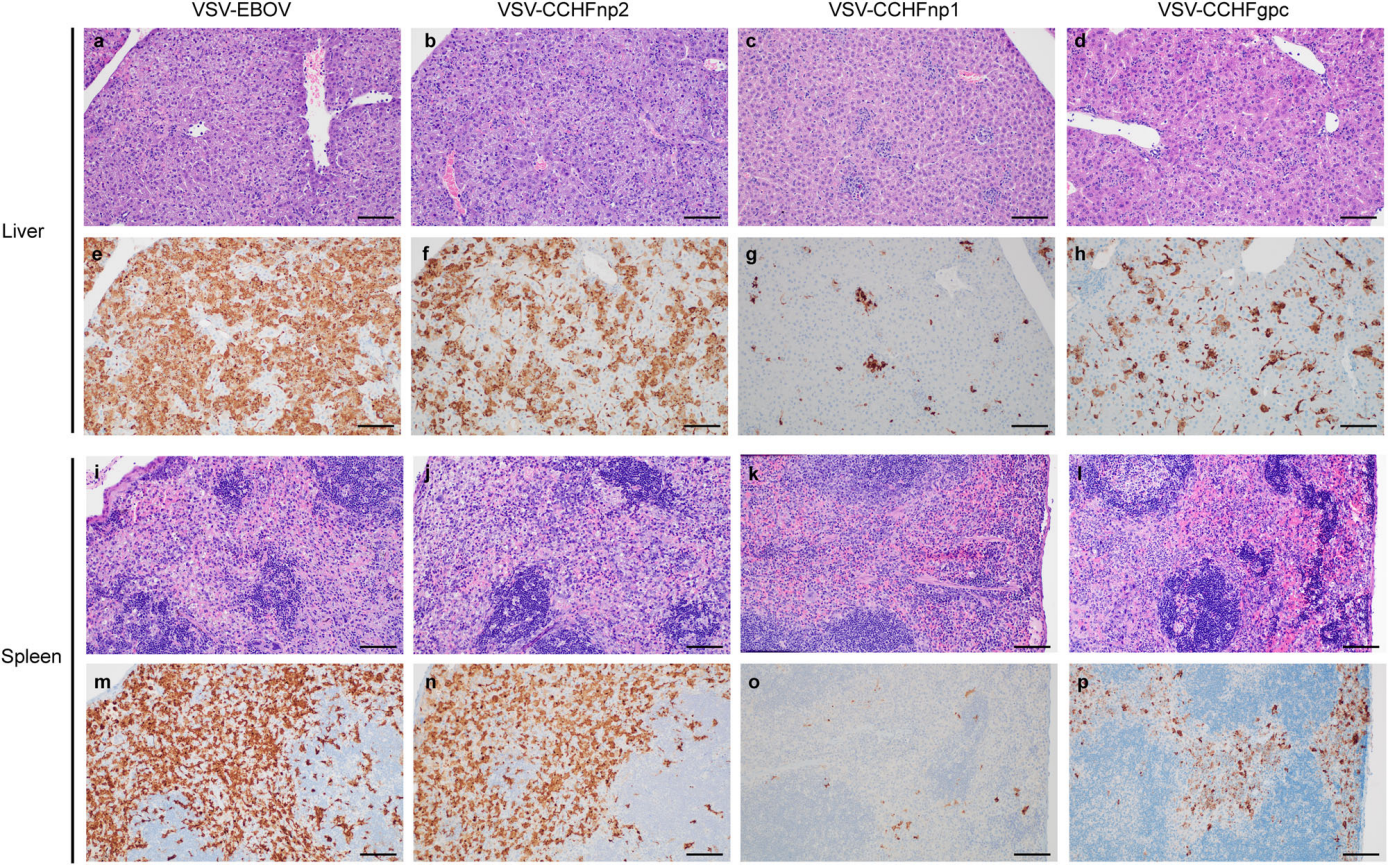
VSV-CCHFnp1 vaccine reduces liver and spleen pathology. Six-week-old C57BL/6J mice were vaccinated intraperitoneally (IP) with 1 × 10^4^ PFU VSV-CCHFnp1, VSV-CCHFnp2, VSV-CCHFgpc, or VSV-EBOV on day -28 relative to challenge. On day 0, mice were treated IP with MAR1-5A3 to block type I IFN receptor signaling and challenged IP with 100 TCID_50_ CCHFV strain UG3010. Liver tissue (**a**–**h**) and spleen tissue (**i**–**p**) were collected from euthanized animals for Hematoxylin and Eosin staining and CCHFV anti-NP immunohistochemistry at day 5 post challenge. All photomicrographs taken at 200x; scale bar = 100 µm.

Histological lesions and anti-CCHFV-NP immunoreactivity were assessed in liver and spleen, key tissues for CCHFV replication and pathology, to better characterize protection conferred by the recombinant VSV vaccine candidates (Supplementary Table 1). Liver lesions in the VSV-EBOV and VSV-CCHFnp2 groups were characterized by multifocal to coalescing random hepatocellular degeneration and necrosis (Fig. 4a, b). Lesions in the VSV-CCHFnp1 group were ~50–75% fewer and more inflammatory rather than necrotic as in the VSV-EBOV and VSV-CCHFnp2 groups (Fig. 4c). These VSV-CCHFnp1 group lesions were multifocal and randomly distributed and consisted of macrophages and neutrophils forming clusters within sinusoids. The VSV-CCHFgpc group’s lesions were also multifocal and randomly distributed with inflammation and scattered necrosis throughout the sinusoids (Fig. 4d). Diffuse anti-CCHFV immunoreactivity was observed in the VSV-EBOV group, with ~25% less observed in the VSV-CCHFnp2 group (Fig. 4e, f). Whereas in the VSV-CCHFnp1 group, immunoreactivity was reduced by ~50% and was focused on inflamed areas and within Kupffer cells (Fig. 4g). The VSV-CCHFgpc group demonstrated less anti-CCHFV immunoreactivity than the VSV-EBOV and the VSV-CCHFnp2 groups but ~50% more than the VSV-CCHFnp1 group (Fig. 4h).

For the spleen, the white pulp was depleted, and the red pulp contained abundant necrotic cellular debris in the VSV-EBOV and VSV-CCHFnp2 vaccinated animals (Fig. 4i, j). The VSV-CCHFnp1 group showed mice with normal appearing spleens (Fig. 4k) and mice with loss of the white pulp. The VSV-CCHFgpc group also showed a loss of the white pulp and necrotic debris were scattered throughout the red pulp (Fig. 4l). Anti-CCHFV immunoreactivity was diffusely abundant throughout the red pulp and the white pulp of VSV-EBOV and VSV-CCHFnp2 vaccinated mice (Fig. 4m, n) while anti-CCHFV immunoreactivity for the VSV-CCHFnp1 was reduced by over 75% and limited to punctate foci within individual cells scattered throughout the red pulp and much less in the white pulp (Fig. 4o). A moderate amount of anti-CCHF immunoreactivity was observed in macrophages and debris within the red pulp for the VSV-CCHFgpc group (Fig. 4p) (~50% more than the VSV-CCHFnp1 group). Cumulatively, the VSV-CCHFnp1 conferred the greatest protection against a heterologous CCHFV challenge, suggesting that the more conserved CCHFV-NP antigen elicits an immune response capable of protecting against genetically diverse strains of CCHFV.

### VSV-CCHFgpc vaccination prevents weight loss and reduces liver pathology in homologous challenged animals

**Fig. 5:**
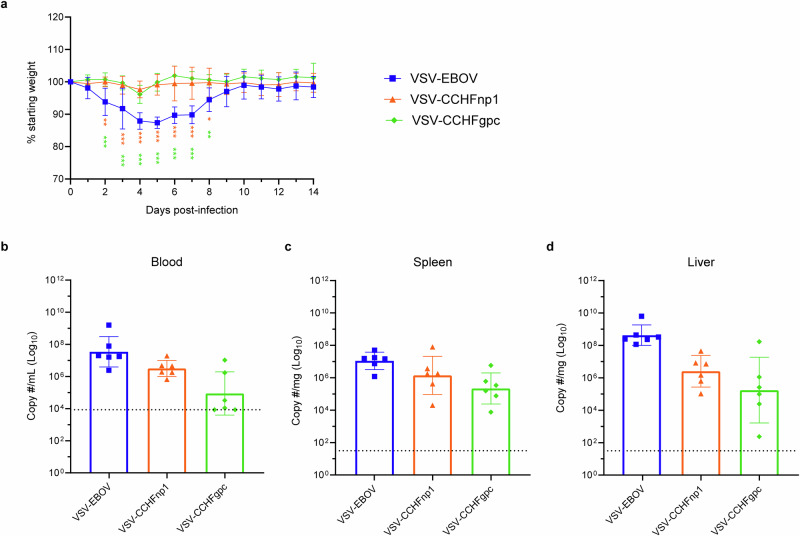
VSV-CCHFgpc vaccines prevents weight loss and reduces viral RNA load following challenge with the CCHFV strain Hoti. Six-week-old C57BL/6J mice were vaccinated intraperitoneally (IP) with 1 × 10^4^ PFU VSV-CCHFnp1, VSV-CCHFgpc, or VSV-EBOV on day -28 relative to challenge. On day 0 mice were treated IP with MAR1-5A3 to block type I IFN receptor signaling and challenged IP with 100 TCID_50_ CCHFV strain Hoti. **a** Mice were weighed daily. *N* = 8 mice per group. Statistical significance compared to VSV-EBOV vaccinated animals is shown. **b**–**d** Viral RNA in indicated tissues at day 5 post challenge was quantified by RT-qPCR. *N* = 6 mice per group. Statistical comparisons calculated using two-way ANOVA with Sidak’s multiple comparisons test (**a**) or one-way ANOVA with Tukey’s multiple comparisons test (**b**–**d**) and results are indicated as *p < 0.05, **p < 0.01, ***p < 0.001, and ****p < 0.0001. Comparisons with *p* values >0.05 were not displayed. Data shown as geometric mean plus standard deviation.

Several vaccines for CCHFV utilizing the GPC, including a VSV-CCHFgpc^36^ have shown protection, in contrast to our findings. However, many of these studies utilized a homologous viral challenge, and incomplete protection was observed when mice vaccinated with a DNA-based GPC vaccine were challenged with a heterologous strain^38^. Therefore, we hypothesized that our recombinant VSV-CCHF vaccine candidates would confer more potent protection against a homologous CCHFV strain Hoti challenge. Since VSV-CCHFnp2 did not show good protection against the CCHFV strain UG3010, we did not include this recombinant VSV vaccine candidate in this experiment. Animals were immunized (IP) with 10^4^ PFU of VSV-CCHFnp1 or VSV-CCHFgpc vaccine candidates. Control group animals received 10^4^ PFU of the VSV-EBOV vaccine. All vaccinated animals produced either CCHFV-NP (VSV-CCHFnp1) or Gc and GP38-specific antibodies (VSV-CCHFgpc) (Supplementary Fig. 5). On day 0, all animals were treated with MAR1-5A3 antibody and were subsequently challenged (IP) with 100 TCID_50_ of the CCHFV strain Hoti. Following the viral challenge, mice weights were monitored daily. In contrast to mice vaccinated with the VSV-EBOV vaccine that lost 10–16% of their pre-infection weights, VSV-CCHFnp1 and VSV-CCHFgpc vaccinated animals showed no weight loss after CCHFV infection (Fig. 5a), demonstrating that both VSV-CCHFnp1 and VSV-CCHFgpc confer protection against homologous challenge. In contrast to the CCHFV strain UG3010 challenge, infection of MAR1-5A3-treated animals with 100 TCID_50_ of CCHFV Hoti is non-lethal, and all animals recovered from CCHFV infection.

To examine the extent of protection following CCHFV challenge, we collected blood, spleen, and liver samples on day 5 PI and evaluated viral RNA genome copies by RT-qPCR. CCHFV-specific viral RNA was detected in all challenged groups, although viral loads trended lower in vaccine groups compared to VSV-EBOV-vaccinated animals (Fig. 5b–d). VSV-CCHFgpc-vaccinated animals displayed lower but not statistically significant viral RNA levels in the blood, liver, and spleen compared to the VSV-CCHFnp1 group (Fig. 5b–d), and infectious CCHFV was not detected in any samples, independent of vaccination group. This result demonstrates that all animals developed viremia following viral challenge despite the presence of a robust antibody response.

**Fig. 6:**
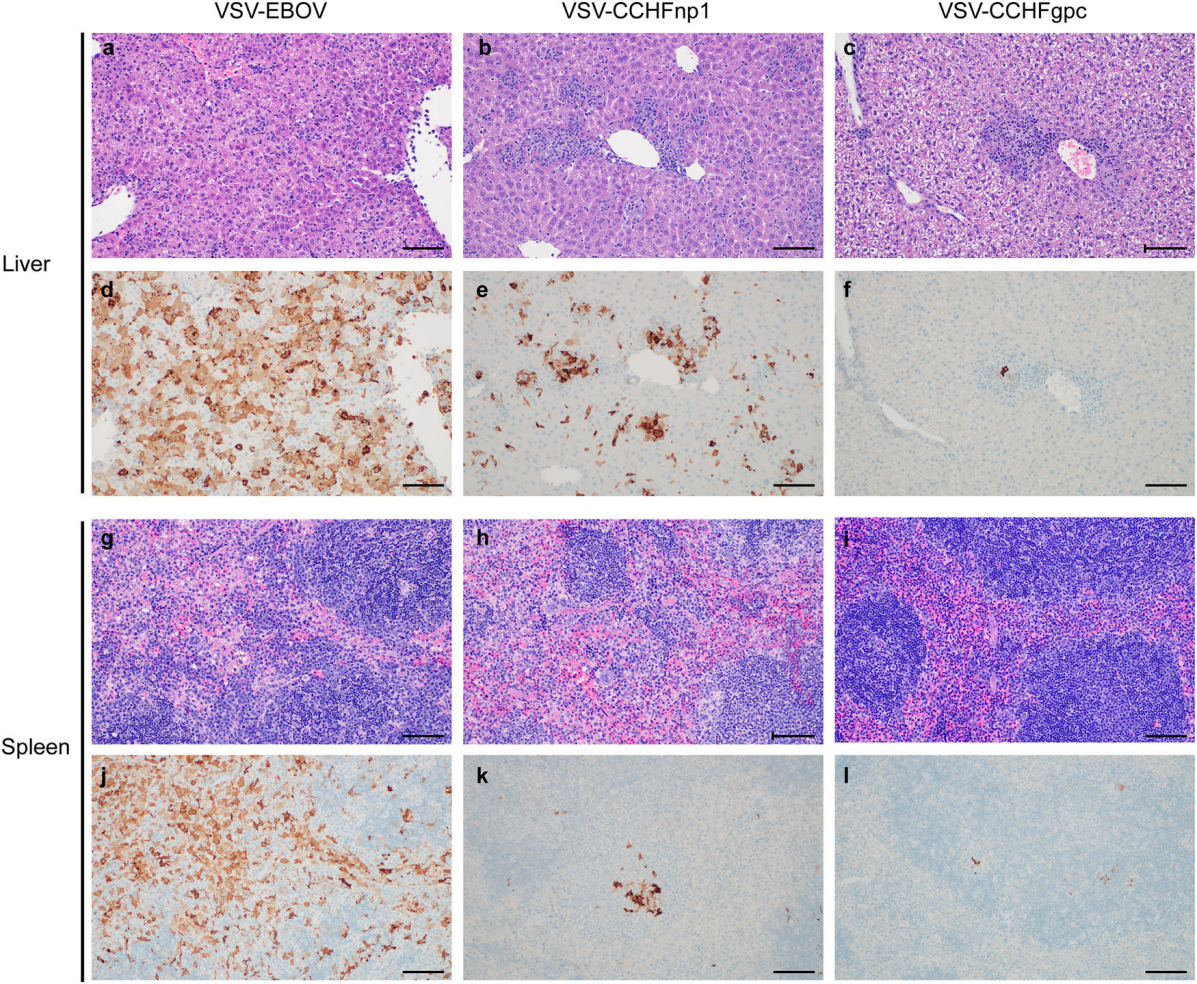
VSV vaccines reduce liver pathology. Six-week-old C57BL/6J mice were vaccinated intraperitoneally (IP) with 1 × 10^4^ PFU VSV-CCHFnp1, VSV-CCHFnp2, VSV-CCHFgpc, or VSV-EBOV on day -28 relative to challenge. On day 0 mice were treated IP with MAR1-5A3 to block type I IFN receptor signaling and challenged IP with 100 TCID_50_ CCHFV strain Hoti. Day 5 post challenge Liver tissue (**a**–**f**) and spleen tissue (**g**–**l**) were collected on day 5 post challenge for Hematoxylin and Eosin staining and CCHFV anti-NP immunohistochemistry. All photomicrographs taken at 200x; scale bar = 100 µm.

To further determine specific differences in protection conferred by the recombinant VSV-CCHF vaccine candidates, we next assessed histologic lesions in liver and spleen collected on day 5 PI (Supplementary Table 2). Histological lesions in the liver consisted predominantly of macrophages and neutrophils forming clusters within sinusoids. Marked intra-sinusoidal inflammation and cellular swelling as well as multifocal to coalescing random hepatocellular degeneration and necrosis was observed in the VSV-EBOV group (Fig. 6a). Similar but often more coalesced lesions occurred in the VSV-CCHFnp1 group (Fig. 6b). VSV-CCHFgpc group’s lesions were also multifocal, coalescing and randomly distributed but with less necrosis than in the VSV-EBOV and VSV-CCHFnp1 groups (Fig. 6c). Near diffuse anti-CCHFV immunoreactivity was observed in hepatocytes and Kupffer cells in the VSV-EBOV group (Fig. 6d) contrasted to multifocal and coalescing but not diffuse immunoreactivity in the VSV-CCHFnp1 group (Fig. 6e) and scant immunoreactivity in the VSV-CCHFgpc group (Fig. 6f).

Significant histologic lesions were not noted in spleens from the three groups (Fig. 6g–i), however, proliferation of mononuclear cells, presumably macrophages, did occur at the margin of the red and white pulp of the VSV-EBOV group. Anti-CCHFV immunoreactivity was greatest and nearly diffuse throughout the red pulp of the VSV-EBOV group (Fig. 6j), with 75 to 90% less immunoreactivity in both the VSV-CCHFnp1 and VSV-CCHFgpc groups, respectively (Fig. 6k, l). Overall, the VSV-EBOV group developed lesions consistent with CCHFV infection in mice, confirming the absence of protection, while the VSV-CCHFnp1 and VSV-CCHFgpc presented with fewer lesions and CCHFV immunoreactivity.

Overall, in contrast to our heterologous challenge model, both recombinant VSV vaccine candidates mediate protection against homologous CCHFV challenge in a non-lethal mouse model. As expected, based on sequence diversity in the CCHFV-GPC, VSV-CCHFVgpc performed better against homologous than heterologous CCHFV challenge.

## Discussion

In this study, we evaluated the protective efficacy of single-dose recombinant VSV-CCHF vaccine candidates targeting the NP and GPC as immunogens. VSV-CCHFVnp2 completely failed in protection against lethal heterologous CCHFV challenge. In contrast, a single dose vaccination with VSV-CCHFnp1 uniformly protected mice against lethal heterologous CCHFV challenge, while the VSV-CCHFgpc vaccine only showed partial protection. Subsequent efficacy testing in a non-lethal homologous CCHFV challenge model, both recombinant VSV vaccine candidates performed similarly in reducing clinical signs and pathology in key target tissues.

Truncation of the Gc C-terminal region has been reported to facilitate VSV pseudotyping with CCHFV-GPC^35,36^ and the CCHFV-GPC_del53 mutant grew to highest viral titers among all CCHFV-GPC mutants investigated^35^. Even though we generated recombinant VSV with CCHFV-GPC_del53 (VSV-CCHFgpc) relatively easily, viral titers for our recombinant VSV-CCHFgpc vaccine candidate were rather low. To enhance virus growth, we passaged the VSV-CCHFgpc in Vero E6 cells, which led to two amino acid mutations (R520K and N759T) and a synonymous mutation at nucleotide position 1122 (S374S). All three mutations occurred in the region encoding the PreGn, with one mutation (R520K) positioned within the subtilisin kexin isozyme-1/site-1 protease cleavage site that generates mature Gn. The consensus motif for subtilisin kexin isozyme-1 (SKI-1)/site-1 protease cleavage is typically described as (R/K)X-(hydrophobic amino acid), where R/K represents either arginine or lysine, X is any amino acid, and the cleavage occurs after the hydrophobic residue^39^. SKI-1 cleavage is essential for the proper function of the CCHFV glycoproteins. Herein, we show that VSV-CCHFgpc is infectious, demonstrating that the glycoproteins are functionally expressed, mediating virus entry and fusion. Another group reported rescuing a recombinant VSV expressing a different CCHFV strain GPC that contained an L518V mutation^36^ and not violating the SKI-1 consensus motif suggesting that rescue of recombinant VSV expressing CCHFV-GPC requires modification but not abolishment of the motif. Interestingly, despite the sequence flexibility of the consensus SKI-1 motif, the sequence conservation of the motif in CCHFV, 520-R(K/R)LL within a broader conserved amino acid 518–526 region suggests that additional selective pressures beyond SKI-1 cleavage maintain this sequence in authentic CCHFV. Further, two independent rescues of recombinant VSV utilizing GPCs from two different CCHFV strains and both with mutations in the motif suggest that selective pressures acting on this motif are distinct between authentic CCHFV and VSV-CCHFgpc particles. Further studies will be needed to understand how these mutations impact CCHFV-GPC polyprotein processing.

Synonymous mutation S374S occurred in the vicinity of S368T, another previously reported mutation^36^, suggesting that this genomic region is also under distinct selective pressure between CCHFV and VSV-CCHFgpc. Yet our synonymous mutation suggests the selective pressures may occur at the RNA sequence rather than the protein level. The N759T mutation has not been reported before, and its potential influence has not been determined. The precise mechanisms by which partial deletion of the Gc C-terminal region and additional mutations in the CCHFV-GPC enhances recombinant virus production is unclear, but while CCHFV buds into the ER/Golgi^5^, VSV buds from the cytoplasmic membrane^40^. Thus, these mutations may promote interaction with the VSV-M, known to localize at the plasma membrane and to promote VSV assembly and budding of virions from host cells^41,42^. The effects of these mutations on immunogenicity need to be studied in future experiments, but since our VSV-CCHFgpc is replication-competent, the mutated glycoproteins retain their binding and entry functions.

Our findings show that VSV-CCHFgpc candidate vaccine prevented weight loss and reduced liver pathology against a homologous challenge but could only confer ~40% protection against a heterologous challenge demonstrating that the high-sequence diversity of the CCHFV-M segment can lead to vaccine failure. This is an important consideration for CCHFV vaccines, as the development of vaccines tailored to each strain of CCHFV is impractical. Since CCHFV is endemic in regions with limited healthcare resources, it is unlikely that a regional vaccine approach for each CCHFV clade would find sufficient funding to advance the multiple vaccines through clinical trials. Partial protection conferred by the VSV-CCHFgpc vaccine candidate in this study against a heterologous CCHFV challenge correlates with our previous findings with the repRNA CCHF GPC vaccine^17^. A GPC DNA-vaccine also exhibited incomplete protection against heterologous challenge^38^, cumulatively suggesting the high genetic diversity of CCHFV in the GPC may enable escape of vaccine-elicited immunity. However, in contrast to our findings with a repRNA and DNA-based GPC vaccine which elicited T cell but not antibody responses, here the VSV-CCHFgpc vaccine candidate induced both neutralizing IgG and IFN-γ T cell response in vaccinated animals that resulted in better protection against a homologous than heterologous CCHFV challenge. We also detected GP38-specific antibodies in VSV-CCHFgpc vaccinated mice. The non-structural protein GP38 may be an important component of GPC-based vaccines^38,43,44^, but GP38-specific antibodies may be strain-specific^38^.

The GP38 of CCHFV strain Hoti and UG3010 GP38 share only 71% amino acid similarity. Recently, a DNA-based GPC vaccine was shown to require T cell responses for protection^45^. Further, it is unclear if the CCHFV-specific T-cells elicited by VSV-CCHFgpc vaccination are virus strain-specific or if those T-cells could recognize strain UG3010-infected cells. We have previously shown that infection of naïve C57BL6/J mice resulted in the majority of CCHFV- specific CD8 T-cells targeting a single, highly conserved Gc epitope^46^. The minimal Gc epitope was identified as an 8-mer 1148-YSPVFEYL^46^. This epitope is in peptide pool 10, the same pool against which we measured the strongest response in VSV-CCHFgpc vaccinated mice, suggesting this conserved peptide may also be targeted in vaccinated mice. Vaccinating mice with the repRNA CCHFV-GPC vaccine induced IFN-γ T cell responses directed against GPC peptide pools 9 and 10^17^, suggesting that other vaccine platforms target similar epitopes. However, further study will be needed to precisely map the T cell epitopes targeted by VSV-CCHFgpc-elicited T-cells. Previously, high-dose (10^7^ PFU) prime-only and prime-boost vaccination with a recombinant VSV expressing CCHFV-GPC vaccine was reported to be protective in a CCHF STAT1^−/−^ mice model against lethal heterologous CCHFV challenge^36^. This discrepancy may be explained by the sensitivity of VSV replication to the inhibitory effects of IFN signaling^47,48^; thus, the STAT1^−/−^ mice model is expected to better promote replication of recombinant VSV vaccines than in immunocompetent C57BL6/J mice used in our study. The higher dose of recombinant VSV vaccine used in that study may have also promoted greater immune responses.

Our findings with VSV-CCHFnp1 confirm the protective effects of immune responses directed against the CCHFV-NP. The CCHFV-NP has previously been investigated as a target antigen for CCHF vaccine development^17,19,49–52^. These studies in animals suggest that the vaccine platform influences CCHFV-NP vaccine efficacy. Uniform protection has been reported with RNA vaccines^17,50^ while viral vectored vaccines have either failed to protect^51^ or conferred partial protection^52^. Here, we observed a robust antibody response to NP, characterized by a dominant IgG2c subclass similar to NP vaccine studies based on the repRNA^17^ and the CCHF replicon particle (VRP)^53^ but different to a dominant IgG1 response observed with bovine herpesvirus type 4 (BoHV-4) and adenovirus type 5 based vaccines (Ad5-N)^19^. Comparing the immunogenicity of these vaccines is challenging because of differences in study designs, but the mouse strain used could also influence the resultant immune profile. The Th1 response observed with the repRNA, VRP, and our present study is consistent with the dominant immunological profile induced by C57BL/6J mice, while the Th2 response with the BoHV-4 and Ad5-N-based vaccines is compatible with the dominant immune response in BALB/c mice. We have recently shown that antibodies against the CCHFV-NP protect through engagement of the cytoplasmic Fc-receptor tripartite motif-containing protein 21 (TRIM21)^54^, and future studies have to demonstrate if VSV-CCHFnp1 may protect through similar mechanisms.

To be replication-competent, the recombinant VSV vaccines expressing the CCHFV nucleoprotein need to express a functional glycoprotein to mediate receptor binding and entry. We used an in-house VSV-EBOV plasmid expressing the EBOV-GP for these purposes. Based on a previous efficacy study with VSV-EBOV and lethal EBOV challenge^31^, we had hypothesized that deleting the immunodominant regions of the EBOV-GP, which are located in the mucin-like domain and glycan cap^32,34,55^, would divert immune responses towards the CCHFV-NP. Here, we did not confirm this hypothesis as VSV-CCHFVnp1 expressing the full-length EBOV-GP mediated higher humoral immune responses and resulted in uniform protection against lethal CCHFV challenge. Interestingly, previous studies evaluating similar VSV-based Kyasanur Forest disease virus vaccines did not result in a lack of immunity and protection when comparing the use of full-length versus truncated EBOV-GP^56^. Differences in outcome could be explained by the choice of antigens and the challenge models, and future studies need to look closer into those factors.

Since VSV-CCHFnp1 showed complete but non-sterile protection against heterologous challenge, improvement may be feasible. A combined recombinant VSV vaccine expressing both GPC and NP may result in even greater protective immunity by eliciting protective immunity to multiple CCHFV antigens. Therefore, a combined recombinant VSV approach expressing both GPC and NP may optimize the here-presented recombinant VSV-based CCHFV vaccine approach. This could be achieved through blending both VSV-CCHFgpc and VSV-CCHFnp1 vaccine candidates. A similar approach had been successful previously for adenoviral and VSV-based vaccines against SARS-CoV-2^57^ and filoviruses^58^. Another strategy would be to generate a single recombinant VSV expressing both antigens as has been done for filoviruses and SARS-CoV-2 with VSV, adenoviral and modified vaccinia Ankara (MVA) platforms^59–62^. A third option could be a prime-boost approach immunizing sequentially with two recombinant VSV vaccine candidates, in this case VSV-CCHFgpc followed by VSV-CCHFVnp1 or vice versa. VSV vector immunity is likely not a big concern, as a similar approach has been used before^63^. Thus, multiple strategies for future improvement of the VSV platform can be considered. In addition, recombinant VSV vaccine candidates can be combined with other vaccine platforms to optimize protection in a blended or prime-boost strategy.

Our study has important limitations. First, the non-lethal animal model we used to evaluate vaccine efficacy against homologous CCHFV challenge is less optimal and may have to be replaced in the future with a lethal animal model to better reflect the performance of the recombinant VSV-based CCHF vaccine candidates. Second, truncation of the C-terminal domain of the Gc facilitated the generation of recombinant VSV-CCHFgpc and did not abolish binding and entry functions of the CCHFV glycoproteins. However, the effect of this modification on the efficiency of Gc protein processing, folding, interaction with the Gn, and ultimately, the function of the surface glycoproteins remains unknown. Third, the VSV-CCHFnp1 vaccine shows great potential as a CCHFV vaccine candidate but may not be readily accepted due to the inclusion of the EBOV glycoprotein. This, however, did not stop the development of similar VSV vaccine candidates for other pathogens from the flavivirus, orthomyxovirus, and paramyxovirus families^56,64,65^. Recently, a live-attenuated recombinant VSV expressing the EBOV and Nipah virus glycoproteins moved into clinical trials^66^. Fourth, the VSV platform may raise safety concerns as a live-attenuated vaccine, especially neurovirulence. Rodent models have generated inconsistency of neurovirulence patterns with different recombinant VSV candidates, favoring neurovirulence testing in nonhuman primate models. Neurovirulence testing of several recombinant VSV vaccine candidates in nonhuman primates revealed favorable data indicating that the vaccines will have an acceptable safety profile also for humans^67–69^. In addition, most of the recombinant VSV vaccine candidates lack the VSV glycoprotein, an important protein associated with VSV pathogenicity, including neurovirulence^70^. Furthermore, the use of VSV-EBOV in humans during Ebola outbreaks has generated little concern regarding side effects^71^. Fifth, there is concern about the potential for recombinant VSV vaccines causing spillover into animal populations, especially livestock. Studies with the VSV-EBOV vaccine, however, show that vaccine virus shedding was minimal in pigs^72^, suggesting the risk of spillover infections in animals is low^72^. Further studies looking at the spillover potential of recombinant VSV vaccines into livestock and other animal species are needed for more precise risk assessment.

In summary, we have developed and characterized recombinant VSV expressing the CCHFV-NP and GPC as vaccine candidates. The VSV-CCHFnp1 vaccine candidate protected all animals against lethal heterologous CCHFV challenge in a transiently immune-suppressed CCHF mice model, while the VSV-CCHFgpc vaccine candidate afforded partial protection against lethal heterologous CCHFV challenge. Together, these data suggest that (1) immunity to the CCHFV-NP or GPC can protect against CCHFV challenge, (2) live-attenuated recombinant VSV expressing CCHFV-NP elicits complete protective immunity after a single immunization, (3) recombinant VSV vaccine candidate utilizing the CCHFV glycoproteins may require tailoring to match circulating strains of CCHFV to generate regionally strain-specific vaccines. At this point, we propose to move ahead with VSV-CCHFVnp1 as the most promising vaccine candidate.

## Methods

### Biosafety and ethics

All infectious work with CCHFV was conducted at biosafety level 4 (BSL-4) following operating procedures approved by the Institutional Biosafety Committee of the Rocky Mountain Laboratories, Division of Intramural Research, National Institute of Allergy and Infectious Diseases, National Institutes of Health (Hamilton, MT, USA). Animal experiments were approved by the Rocky Mountain Laboratories Animal Care and Use Committee. All animal procedures were carried out by trained and certified personnel in accordance with the guidelines of the Association for Assessment and Accreditation of Laboratory Animal Care, International and the Office of Laboratory Animal Welfare. Mice were group-housed in HEPA-filtered cage systems enriched with nesting material. Commercial food and water were available ad libitum.

### Cells and viruses

Vero E6 cells were maintained in Dulbecco’s modified Eagle’s medium (DMEM) (Sigma-Aldrich, St. Louis, MO) supplemented with 10% fetal bovine serum (FBS) (Wisent Inc., St. Bruno, Canada), 2 mM l-glutamine, 50 U/mL penicillin, and 50 μg/mL streptomycin (all Thermo Fisher Scientific, Waltham, MA). Baby hamster kidney cells expressing the T7 polymerase (BHK-T7) were grown at 37 °C and 5% CO_2_ in minimum essential medium (MEM) containing 10% tryptose phosphate broth (both Thermo Fisher Scientific), 5% FBS, 2 mM l-glutamine, 50 U/mL penicillin, and 50 μg/mL streptomycin. VSV-wt, VSV-EBOV, and VSV-EBOVΔGCΔMLD were previously generated in-house^31,73^. CCHFV strain UG3010 was originally provided by Eric Bergeron, Centers for Disease Control and Prevention. CCHFV Hoti was provided through the European Virus Archive, https://www.european-virus-archive.com/^74^.

### Generation of vaccine candidates

Recombinant VSV vaccine candidates were prepared using sequences for the NP and a codon-optimized GPC from the CCHFV strain Hoti (Genbank Accession numbers: MH483984 and MH483985, respectively). The NP was ligated into the VSV-EBOV or VSV-EBOVΔGCΔMLD plasmids^31^ using *PacI* and *AscI* restriction enzyme sites (NEB) while the GPC was cloned into the pATX-VSVΔG-XN2 plasmid^73^ at the *Avrll* and *MluI* restriction sites (NEB). The replication-competent recombinant VSV vaccine candidates were recovered from the plasmid on BHK-T7 cells as previously described^75^. The complete genome sequences of the generated vaccine candidates were confirmed by Next-generation sequencing. The titer of virus stocks was determined using a standard plaque assay on Vero E6 cells. Briefly, Vero E6 cells grown to 100% confluence in six-well plates were infected in triplicate with recombinant VSV vaccine candidates serially diluted from 10^−1^ to 10^−8^. Following incubation at 37 ^0^C for 1 h, the virus inoculum was replaced with a 1:1 mixture of 2% LMP agarose (Invitrogen, Carlsbad, CA) in 2 × MEM/4% FBS (Thermo Fisher Scientific). The agarose was allowed to solidify at room temperature. Subsequently, the plates were incubated at 37 ^0^C for 72 h. The plaques were stained with crystal violet and vaccine titers were calculated in PFU/mL.

### VSV growth kinetics

Confluent Vero E6 cells were infected in triplicate with the recombinant VSV vaccine candidates and VSV-wt (see above) at a multiplicity of infection of 0.01. After 1 h incubation, infected cells were washed three times with plain DMEM, and 2% DMEM added to the wells. Supernatant samples were collected at 0, 6, 12, 24, 36, 48, and 72-h post infection and stored at −80 ^0^C. Virus was titrated on Vero E6 cells using the TCID_50_ assay previously described^76^.

### Immunofluorescence assay

Vero E6 cells were infected with the recombinant VSV vaccine candidates and VSV-wt at an MOI of 0.01. At 24 h post infection, the cells were fixed with 2% paraformaldehyde (PFA) and permeabilized using 0.05% Triton X-100 in PBS. Cells were first stained with CCHFV-NP specific sera raised in rabbits, 11E7 monoclonal antibody (BEI resources), anti-EBOV-GP 12/1.1 (kindly provided by Ayato Takada, Hokkaido University, Sapporo, Japan), or anti-VSV-M (23H12, Kerafast Inc., Boston, MA). Secondary staining was performed using an Alexa Fluor 488 goat anti-mouse IgG (Invitrogen). Images were taken using the Revolve fluorescent cell imager (ECHO, San Diego, CA).

### Western blot analysis

Tissue culture supernatant samples and cell lysates derived from Vero E6 cells infected with the recombinant VSV vaccine candidates and VSV-wt were mixed with sodium dodecyl sulfate-polyacrylamide (SDS) gel electrophoresis sample buffer containing 20% β-mercaptoethanol and heated to 99 °C for 10 min. Analysis of the samples was performed as described elsewhere^31^. The CCHFV-NP and Gc protein blots were probed with NP specific sera raised in rabbits and 11E7 monoclonal antibody (BEI resources), respectively. While the EBOV glycoprotein and VSV-M protein blots were probed with the following mouse monoclonal antibodies: anti-EBOV-GP 12/1.1 (kindly provided by Ayato Takada, Hokkaido University, Sapporo, Japan), and anti-VSV-M (23H12; Kerafast Inc., Boston, MA). Secondary staining was performed with anti-rabbit or anti-mouse IgG (Jackson ImmunoResearch, West Grove, PA). Detection was performed using SuperSignal West Pico chemiluminescent substrate (Thermo Fisher Scientific), and images were captured with the iBright^TM^ CL1500 imaging system (Thermo Fisher Scientific).

### Vaccine efficacy studies

Groups of 20 C57BL6/J mice (6–8 weeks of age; males and females) were anaesthetized with isoflurane and vaccinated IP on day -28 with 1 × 10^4^ PFU of VSV-EBOV, VSV-CCHFnp1, VSV-CCHFnp2, or VSV-CCHFgpc. Vaccination appeared well tolerated, and no adverse events attributable to the vaccine were observed during the studies. On day 0, mice (*n* = 6) in each group were euthanized for blood sample collection. The remaining mice received 2.5 mg/mouse of MAR1-5A3 (Leinco) and were challenged with 100 TCID_50_ of CCHFV strain UG3010 or CCHFV strain Hoti via the IP route. On day 5 post infection (PI), mice (*n* = 6) in each group were euthanized for blood and tissue sample collection. The remaining mice were monitored daily for 14 days for clinical disease and survival outcomes. Mice were euthanized via isoflurane overdose followed by cervical dislocation at the time points indicated or when meeting euthanasia criteria according to protocols approved by Rocky Mountain Laboratories Institutional Animal Care and Use Committee. No sex-specific differences in vaccine immunogenicity or disease outcome were observed.

### Total RNA extraction and quantitative reverse transcription PCR (RT-qPCR)

Liver, spleen, and whole blood specimens were collected at day 5 PI. Approximately 30 mg of tissue specimens were homogenized with a steel bead in RLT buffer supplemented with 10 µL/ml β-mercaptoethanol using the TissueLyser II (Qiagen). After a 10-min spin at 8000 rpm, the supernatant was added to an equal volume of 70% ethanol. RNA from blood and tissues was extracted using Qiagen Qiamp viral RNA mini-isolation kit and Qiagen RNeasy mini-isolation kit, respectively, as per the manufacturer’s instructions. A one-step RT-qPCR was performed using primers and probes specific for the CCHFV-NP^17^ as previously described.

### Tissue infectious virus titers

Infectious CCHFV from liver, spleen, and whole blood specimens was quantified on SW-13 cells as previously described^77^. TCID_50_ values were then calculated using the Reed and Muench method^78^.

### ELISA

Total CCHFV IgG and IgG subclasses specific antibodies were detected using an in-house ELISA using whole CCHFV Hoti antigen or recombinant GP38 or Gn or Gc antigen as previously described^74,79^. Total EBOV-GP IgG-specific antibodies were evaluated using recombinant Ebola virus GP antigen (IBT Bioservices). Nunc MaxiSorp 96-well plates (Thermo Fisher Scientific) were coated with 100 µl of CCHFV whole virion-antigen or CCHFV-GP38 or Gn protein with His-tag or Gc protein with sheep Fc-tag (Native antigen) or Ebola virus glycoprotein minus the transmembrane domain (IBT Bioservices) at 1 µg/mL and incubated overnight at 4 ^o^C. The following day, plates were washed with phosphate-buffered saline (PBS) containing 0.05% Tween 20 (PBST) and blocked with 1% skim milk in PBST, followed by 1 h incubation at room temperature. Subsequently, a fourfold dilution of mouse sera in PBST starting at 1:100 was added to the plate and incubated for 1 h at room temperature. Detection was performed using horseradish peroxidase-labeled goat anti-mouse total IgG, IgG1, IgG2b, IgG2c, and IgG3 (Southern Biotech) and developed by addition of ABTS substrate solution for 30 min. Absorbance was read at 405 nm using the BioTek Synergy HTX Multimode Reader. Absorbances were plotted on the y-axis and the reciprocal serum dilutions on the x-axis. The limit of detection was calculated as follows: average OD_405_ of the control antigen coated + 3xSD. The cutoff for seropositivity was deemed where the test OD_405_ intersects the limit of detection.

### Virus neutralization assay

Two-fold dilutions in triplicate of sera from vaccinated mice was incubated with 120 TCID_50_ of either CCHFV strain Hoti or UG3010 for 1 h at 37 ^o^C followed by infection of confluent SW-13 cells. Serum toxicity was checked 24 h post infection, and cytopathic effect was read on day 5 PI. Neutralization titers (NT) were described as the highest serum dilution that completely neutralized 120 TCID_50_ of either the CCHFV strain Hoti or UG3010. The initial dilution of sera was 1:10, which was set as the limit of detection.

### IFNγ ELISpot

An IFNγ ELISpot was performed on splenocytes stimulated with peptides derived from the CCHFV Hoti NP and GPC as previously described^17^.

### Histopathology

Tissues were fixed in 10% Neutral Buffered Formalin with two changes, for a minimum of 7 days. Tissues were placed in cassettes and processed with a Sakura VIP-6 Tissue Tek, on a 12-hour automated schedule, using a graded series of ethanol, xylene, and PureAffin. Embedded tissues were sectioned at 5 µm and dried overnight at 42 °C prior to staining. Specific anti-CCHFV immunoreactivity was detected using Rabbit anti-CCHFV nucleoprotein (IBT Bioservices) at a 1:2000 dilution. The Immpress-VR anti-rabbit IgG polymer kit (Vector Laboratories, California) was used as the secondary antibody. The tissues were processed for immunohistochemistry using the Discovery Ultra automated stainer (Ventana Medical Systems) with a ChromoMap DAB kit (Roche Tissue Diagnostics catalog # 760-159).

### Statistical analysis

Statistical analysis was performed using GraphPad Prism version 10.1.0. 316 for Windows (GraphPad Software, San Diego, CA, USA, www.graphpad.com, 20 May 2024). Statistical significance was set at *p* < 0.05.

## Supplementary information

nr-competing-interests

Supplementary files

## Data Availability

Relevant source data for figures are provided within the manuscript and its supporting Information files. Sequence data for recombinant VSV vaccine candidates has been deposited on GenBank (Accession numbers: PV506373-PV506375).

## References

[1] Leventhal, S. S., Wilson, D., Feldmann, H. & Hawman, D. W. A look into Bunyavirales genomes: functions of non-structural (NS) proteins. *Viruses*10.3390/v13020314 (2021). 10.3390/v13020314 PMC7922539 33670641

[2] Altamura, L. A. et al. Identification of a novel C-terminal cleavage of Crimean-Congo hemorrhagic fever virus PreG that leads to generation of an NS protein. *J. Virol.* **81**, 6632–6642 (2007). 17409136 10.1128/JVI.02730-06 PMC1900101

[3] Sanchez, A. J., Vincent, M. J., Erickson, B. R. & Nichol, S. T. Crimean-Congo hemorrhagic fever virus glycoprotein precursor is cleaved by furin-like and SKI-1 proteases to generate a novel 38-kilodalton glycoprotein. *J. Virol.* **80**, 514–525 (2006). 16352575 10.1128/JVI.80.1.514-525.2006 PMC1317557

[4] Sanchez, A. J., Vincent, M. J. & Nichol, S. T. Characterization of the glycoproteins of Crimean-Congo hemorrhagic fever virus. *J. Virol.* **76**, 7263–7275 (2002). 12072526 10.1128/JVI.76.14.7263-7275.2002 PMC136317

[5] Zivcec, M., Scholte, F. E. M., Spiropoulou, C. F., Spengler, J. R. & Bergeron, É. Molecular Insights into Crimean-Congo hemorrhagic fever virus. *Viruses-Basel* **8**, 10.3390/v8040106 (2016). 10.3390/v8040106 PMC4848600 27110812

[6] Tipih, T. & Burt, F. J. Crimean-Congo Hemorrhagic fever virus: advances in vaccine development. *Biores Open Access* **9**, 137–150 (2020). 32461819 10.1089/biores.2019.0057 PMC7247048

[7] Hoogstraal, H. The epidemiology of tick-borne Crimean-Congo hemorrhagic fever in Asia, Europe, and Africa. *J. Med. Entomol.* **15**, 307–417 (1979). 113533 10.1093/jmedent/15.4.307

[8] Gargili, A. et al. The role of ticks in the maintenance and transmission of Crimean-Congo hemorrhagic fever virus: a review of published field and laboratory studies. *Antivir. Res.* **144**, 93–119 (2017). 28579441 10.1016/j.antiviral.2017.05.010 PMC6047067

[9] Spengler, J. R., Bergeron, E. & Rollin, P. E. Seroepidemiological studies of Crimean-Congo hemorrhagic fever virus in domestic and wild animals. *PLoS Negl. Trop. Dis.* **10**, e0004210 (2016). 26741652 10.1371/journal.pntd.0004210 PMC4704823

[10] Bente, D. A. et al. Crimean-Congo hemorrhagic fever: history, epidemiology, pathogenesis, clinical syndrome and genetic diversity. *Antivir. Res.* **100**, 159–189 (2013). 23906741 10.1016/j.antiviral.2013.07.006

[11] Papa, A., Papadimitriou, E. & Christova, I. The Bulgarian vaccine Crimean-Congo haemorrhagic fever virus strain. *Scand. J. Infect. Dis.* **43**, 225–229 (2011). 21142621 10.3109/00365548.2010.540036

[12] Ahata, B. & Akcapinar, G. B. CCHFV vaccine development, current challenges, limitations, and future directions. *Front. Immunol.* **14**, 1238882 (2023). 37753088 10.3389/fimmu.2023.1238882 PMC10518622

[13] Dowall, S. D., Carroll, M. W. & Hewson, R. Development of vaccines against Crimean-Congo haemorrhagic fever virus. *Vaccine* **35**, 6015–6023 (2017). 28687403 10.1016/j.vaccine.2017.05.031 PMC5637709

[14] Kortekaas, J. et al. Crimean-Congo hemorrhagic fever virus subunit vaccines induce high levels of neutralizing antibodies but no protection in STAT1 knockout mice. *Vector Borne Zoonotic Dis.* **15**, 759–764 (2015). 26684523 10.1089/vbz.2015.1855 PMC7643766

[15] Garrison, A. R. et al. A DNA vaccine for Crimean-Congo hemorrhagic fever protects against disease and death in two lethal mouse models. *PLoS Negl. Trop. Dis.* **11**, e0005908 (2017). 28922426 10.1371/journal.pntd.0005908 PMC5619839

[16] Moming, A. et al. Mapping of B-cell epitopes on the N- terminal and C-terminal segment of nucleocapsid protein from Crimean-Congo hemorrhagic fever virus. *PLoS ONE* **13**, e0204264 (2018). 30235312 10.1371/journal.pone.0204264 PMC6147494

[17] Leventhal, S. S. et al. Replicating RNA vaccination elicits an unexpected immune response that efficiently protects mice against lethal Crimean-Congo hemorrhagic fever virus challenge. *Ebiomedicine*10.1016/j.ebiom.2022.104188 (2022). 10.1016/j.ebiom.2022.104188 PMC9335360 35907368

[18] Karaaslan, E. et al. Crimean Congo hemorrhagic fever virus nucleoprotein and GP38 subunit vaccine combination prevents morbidity in mice. *NPJ Vaccines* **9**, 148 (2024). 39143104 10.1038/s41541-024-00931-y PMC11324950

[19] Aligholipour Farzani, T. et al. Bovine herpesvirus type 4 (BoHV-4) vector delivering nucleocapsid protein of Crimean-Congo hemorrhagic fever virus induces comparable protective immunity against lethal challenge in IFNalpha/beta/gammaR-/- mice models. *Viruses*10.3390/v11030237 (2019). 10.3390/v11030237 PMC6466008 30857305

[20] Deyde, V. M., Khristova, M. L., Rollin, P. E., Ksiazek, T. G. & Nichol, S. T. Crimean-Congo hemorrhagic fever virus genomics and global diversity. *J. Virol.* **80**, 8834–8842 (2006). 16912331 10.1128/JVI.00752-06 PMC1563879

[21] Garbutt, M. et al. Properties of replication-competent vesicular stomatitis virus vectors expressing glycoproteins of filoviruses and arenaviruses. *J. Virol.* **78**, 5458–5465 (2004). 15113924 10.1128/JVI.78.10.5458-5465.2004 PMC400370

[22] Mire, C. E. et al. Single injection recombinant vesicular stomatitis virus vaccines protect ferrets against lethal Nipah virus disease. *Virol. J.* **10**, 353 (2013). 24330654 10.1186/1743-422X-10-353 PMC3878732

[23] Ao, Z. J. et al. A recombinant VSV-based bivalent vaccine effectively protects against both SARS-CoV-2 and influenza A virus infection. *J. Virol.*10.1128/jvi.01337-22 (2022). 10.1128/jvi.01337-22 PMC9517730 36069551

[24] Brown, K. S., Safronetz, D., Marzi, A., Ebihara, H. & Feldmann, H. Vesicular stomatitis virus-based vaccine protects hamsters against lethal challenge with Andes virus. *J. Virol.* **85**, 12781–12791 (2011). 21917979 10.1128/JVI.00794-11 PMC3209372

[25] Rose, N. F. et al. An effective AIDS vaccine based on live attenuated vesicular stomatitis virus recombinants. *Cell* **106**, 539–549 (2001). 11551502 10.1016/s0092-8674(01)00482-2

[26] Clarke, D. K. et al. Recombinant vesicular stomatitis virus as an HIV-1 vaccine vector. *Springe. Semin. Immun.* **28**, 239–253 (2006). 10.1007/s00281-006-0042-3 PMC7079905 16977404

[27] Halperin, S. A. et al. Six-month safety data of recombinant vesicular stomatitis virus-Zaire Ebola virus envelope glycoprotein vaccine in a phase 3 double-blind, placebo-controlled randomized study in healthy adults. *J. Infect. Dis.* **215**, 1789–1798 (2017). 28549145 10.1093/infdis/jix189 PMC5853326

[28] Anderson, E. M. & Coller, B. A. Translational success of fundamental virology: a VSV-vectored Ebola vaccine. *J. Virol.* **98**, e0162723 (2024). 38305150 10.1128/jvi.01627-23 PMC10994820

[29] Jones, S. M. et al. Live attenuated recombinant vaccine protects nonhuman primates against Ebola and Marburg viruses. *Nat. Med.* **11**, 786–790 (2005). 15937495 10.1038/nm1258

[30] Marzi, A. et al. VSV-EBOV rapidly protects macaques against infection with the 2014/15 Ebola virus outbreak strain. *Science* **349**, 739–742 (2015). 26249231 10.1126/science.aab3920 PMC11040598

[31] Bhatia, B., Furuyama, W., Hoenen, T., Feldmann, H. & Marzi, A. Ebola virus glycoprotein domains associated with protective efficacy. *Vaccines*10.3390/vaccines9060630 (2021). 10.3390/vaccines9060630 PMC8229685 34200548

[32] Dowling, W. et al. Influences of glycosylation on antigenicity, immunogenicity, and protective efficacy of ebola virus GP DNA vaccines. *J. Virol.* **81**, 1821–1837 (2007). 17151111 10.1128/JVI.02098-06 PMC1797596

[33] Martinez, O., Tantral, L., Mulherkar, N., Chandran, K. & Basler, C. F. Impact of Ebola mucin-like domain on antiglycoprotein antibody responses induced by Ebola virus-like particles. *J. Infect. Dis.* **204**, S825–S832 (2011). 21987758 10.1093/infdis/jir295 PMC3189980

[34] Sanchez-Lockhart, M. et al. Qualitative profiling of the humoral immune response elicited by rVSV-deltaG-EBOV-GP using a systems serology assay, domain programmable arrays. *Cell Rep.* **24**, 1050–1059.e1055 (2018). 30044972 10.1016/j.celrep.2018.06.077

[35] Suda, Y. et al. Analysis of the entry mechanism of Crimean-Congo hemorrhagic fever virus, using a vesicular stomatitis virus pseudotyping system. *Arch. Virol.* **161**, 1447–1454 (2016). 26935918 10.1007/s00705-016-2803-1 PMC7087235

[36] Rodriguez, S. E. et al. Vesicular stomatitis virus-based vaccine protects mice against Crimean-Congo hemorrhagic fever. *Sci. Rep.* **9**, 7755 (2019). 31123310 10.1038/s41598-019-44210-6 PMC6533279

[37] Lindquist, M. E. et al. Exploring Crimean-Congo hemorrhagic fever Virus-induced hepatic injury using antibody-mediated type I interferon blockade in mice. *J. Virol.*10.1128/JVI.01083-18 (2018). 10.1128/JVI.01083-18 PMC6189508 30111561

[38] Suschak, J. J. et al. A CCHFV DNA vaccine protects against heterologous challenge and establishes GP38 as immunorelevant in mice. *Npj Vaccines*10.1038/s41541-021-00293-9 (2021). 10.1038/s41541-021-00293-9 PMC7925670 33654101

[39] Pasquato, A. et al. The proprotein convertase SKI-1/S1P. In vitro analysis of Lassa virus glycoprotein-derived substrates and ex vivo validation of irreversible peptide inhibitors. *J. Biol. Chem.* **281**, 23471–23481 (2006). 16790437 10.1074/jbc.M513675200

[40] Robison, C. S. & Whitt, M. A. The membrane-proximal stem region of vesicular stomatitis virus G protein confers efficient virus assembly. *J. Virol.* **74**, 2239–2246 (2000). 10666254 10.1128/jvi.74.5.2239-2246.2000 PMC111705

[41] Justice, P. A. et al. Membrane vesiculation function and exocytosis of wild-type and mutant matrix proteins of vesicular stomatitis virus. *J. Virol.* **69**, 3156–3160 (1995). 7707543 10.1128/jvi.69.5.3156-3160.1995 PMC189017

[42] Mebatsion, T., Konig, M. & Conzelmann, K. K. Budding of rabies virus particles in the absence of the spike glycoprotein. *Cell* **84**, 941–951 (1996). 8601317 10.1016/s0092-8674(00)81072-7

[43] Scher, G., Bente, D. A., Mears, M. C., Cajimat, M. N. B. & Schnell, M. J. GP38 as a vaccine target for Crimean-Congo hemorrhagic fever virus. *Npj Vaccines*10.1038/s41541-023-00663-5 (2023). 10.1038/s41541-023-00663-5 PMC10199669 37210392

[44] Golden, J. W. et al. GP38-targeting monoclonal antibodies protect adult mice against lethal Crimean-Congo hemorrhagic fever virus infection. *Sci. Adv.* **5**, eaaw9535 (2019). 31309159 10.1126/sciadv.aaw9535 PMC6620094

[45] Golden, J. W. et al. Induced protection from a CCHFV-M DNA vaccine requires CD8(+) T cells. *Virus Res.* **334**, 199173 (2023). 37459918 10.1016/j.virusres.2023.199173 PMC10388194

[46] Rao, D. et al. CD8( + ) T-cells target the Crimean-Congo haemorrhagic fever virus Gc protein to control the infection in wild-type mice. *Ebiomedicine* **97**, 104839 (2023). 37866114 10.1016/j.ebiom.2023.104839 PMC10623175

[47] Fensterl, V. et al. Interferon-induced Ifit2/ISG54 protects mice from lethal VSV neuropathogenesis. *PLos Pathog.*10.1371/journal.ppat.1002712 (2012). 10.1371/journal.ppat.1002712 PMC3355090 22615570

[48] Rubinstein, S., Familletti, P. C. & Pestka, S. Convenient assay for interferons. *J. Virol.* **37**, 755–758 (1981). 6163873 10.1128/jvi.37.2.755-758.1981 PMC171063

[49] Aligholipour Farzani, T. et al. Immunological analysis of a CCHFV mRNA vaccine candidate in mouse models. *Vaccines*10.3390/vaccines7030115 (2019). 10.3390/vaccines7030115 PMC6789841 31527460

[50] Appelberg, S. et al. Nucleoside-modified mRNA vaccines protect IFNAR−/− mice against Crimean-Congo hemorrhagic fever virus infection. *J. Virol.*10.1128/JVI.01568-21 (2022). 10.1128/jvi.01568-21 PMC8826901 34817199

[51] Dowall, S. D. et al. A Crimean-Congo hemorrhagic fever (CCHF) viral vaccine expressing nucleoprotein is immunogenic but fails to confer protection against lethal disease. *Hum. Vaccin. Immunother.* **12**, 519–527 (2016). 26309231 10.1080/21645515.2015.1078045 PMC5049717

[52] Zivcec, M., Safronetz, D., Scott, D. P., Robertson, S. & Feldmann, H. Nucleocapsid protein-based vaccine provides protection in mice against lethal Crimean-Congo hemorrhagic fever virus challenge. *PLoS Negl. Trop. Dis.* **12**, e0006628 (2018). 30011277 10.1371/journal.pntd.0006628 PMC6062107

[53] Scholte, F. E. M. et al. Vaccination with the Crimean-Congo hemorrhagic fever virus viral replicon vaccine induces NP-based T-cell activation and antibodies possessing Fc-mediated effector functions. *Front. Cell Infect. Microbiol.* **13**, 1233148 (2023). 37671145 10.3389/fcimb.2023.1233148 PMC10475602

[54] Leventhal, S. S. et al. Antibodies targeting the Crimean-Congo Hemorrhagic Fever Virus nucleoprotein protect via TRIM21. *Nat. Commun.* **15**, 9236 (2024). 10.1038/s41467-024-53362-7 PMC11511847 39455551

[55] Mitchell, D. A. J. et al. Epitope mapping of Ebola virus dominant and subdominant glycoprotein epitopes facilitates construction of an epitope-based DNA vaccine able to focus the antibody response in mice. *Hum. Vaccin Immunother.* **13**, 2883–2893 (2017). 28699812 10.1080/21645515.2017.1347740 PMC5718802

[56] Bhatia, B. et al. A live-attenuated viral vector vaccine protects mice against lethal challenge with Kyasanur Forest disease virus. *NPJ Vaccines* **6**, 152 (2021). 34907224 10.1038/s41541-021-00416-2 PMC8671490

[57] Dangi, T., Class, J., Palacio, N., Richner, J. M. & Penaloza MacMaster, P. Combining spike- and nucleocapsid-based vaccines improves distal control of SARS-CoV-2. *Cell Rep.* **36**, 109664 (2021). 34450033 10.1016/j.celrep.2021.109664 PMC8367759

[58] Geisbert, T. W. et al. Single-injection vaccine protects nonhuman primates against infection with marburg virus and three species of ebola virus. *J. Virol.* **83**, 7296–7304 (2009). 19386702 10.1128/JVI.00561-09 PMC2704787

[59] O’Donnell, K. L., Gourdine, T., Fletcher, P., Clancy, C. S. & Marzi, A. Protection from COVID-19 with a VSV-based vaccine expressing the spike and nucleocapsid proteins. *Front. Immunol.* **13**, 1025500 (2022). 36353642 10.3389/fimmu.2022.1025500 PMC9638159

[60] Clever, S. et al. Single MVA-SARS-2-ST/N vaccination rapidly protects K18-hACE2 mice against a lethal SARS-CoV-2 challenge infection. *Viruses*10.3390/v16030417 (2024). 10.3390/v16030417 PMC10974247 38543782

[61] Gabitzsch, E. et al. Dual-antigen COVID-19 vaccine subcutaneous prime delivery with oral boosts protects NHP against SARS-CoV-2 challenge. *Front. Immunol.* **12**, 729837 (2021). 34603305 10.3389/fimmu.2021.729837 PMC8481919

[62] Mire, C. E. et al. A single-vector, single-injection trivalent filovirus vaccine: proof of concept study in outbred guinea Pigs. *J. Infect. Dis.* **212**, S384–S388 (2015). 25957964 10.1093/infdis/jiv126 PMC4564539

[63] Marzi, A., Feldmann, F., Geisbert, T. W., Feldmann, H. & Safronetz, D. Vesicular stomatitis virus-based vaccines against Lassa and Ebola viruses. *Emerg. Infect. Dis.* **21**, 305–307 (2015). 25625358 10.3201/eid2102.141649 PMC4313664

[64] Furuyama, W. et al. A single dose of a vesicular stomatitis virus-based influenza vaccine confers rapid protection against H5 viruses from different clades. *NPJ Vaccines* **5**, 4 (2020). 31934358 10.1038/s41541-019-0155-z PMC6954110

[65] de Wit, E. et al. Distinct VSV-based Nipah virus vaccines expressing either glycoprotein G or fusion protein F provide homologous and heterologous protection in a nonhuman primate model. *Ebiomedicine* **87**, 104405 (2023). 36508878 10.1016/j.ebiom.2022.104405 PMC9763366

[66] Public Health Vaccines LLC, C. B. Developing a Nipah virus vaccine. https://nipahvaccine.com (2020).

[67] Monath, T. P. et al. Recombinant vesicular stomatitis vaccine against Nipah virus has a favorable safety profile: model for assessment of live vaccines with neurotropic potential. *PLos Pathog*10.1371/journal.ppat.1010658 (2022). 10.1371/journal.ppat.1010658 PMC9269911 35759511

[68] Clarke, D. K. et al. Live virus vaccines based on a vesicular stomatitis virus (VSV) backbone: standardized template with key considerations for a risk/benefit assessment. *Vaccine* **34**, 6597–6609 (2016). 27395563 10.1016/j.vaccine.2016.06.071 PMC5220644

[69] Mire, C. E. et al. Recombinant vesicular stomatitis virus vaccine vectors expressing filovirus glycoproteins lack neurovirulence in nonhuman primates. *PLoS Negl. Trop. Dis.* **6**, e1567 (2012). 22448291 10.1371/journal.pntd.0001567 PMC3308941

[70] Martinez, I., Rodriguez, L. L., Jimenez, C., Pauszek, S. J. & Wertz, G. W. Vesicular stomatitis virus glycoprotein is a determinant of pathogenesis in swine, a natural host. *J. Virol.* **77**, 8039–8047 (2003). 12829843 10.1128/JVI.77.14.8039-8047.2003 PMC161932

[71] Matz, K. M., Marzi, A. & Feldmann, H. Ebola vaccine trials: progress in vaccine safety and immunogenicity. *Expert Rev. Vaccines* **18**, 1229–1242 (2019). 31779496 10.1080/14760584.2019.1698952

[72] de Wit, E. et al. Safety of recombinant VSV-Ebola virus vaccine vector in pigs. *Emerg. Infect. Dis.* **21**, 702–704 (2015). 25811738 10.3201/eid2104.142012 PMC4378486

[73] Marzi, A. et al. Vesicular stomatitis virus-based Ebola vaccines with improved cross-protective efficacy. *J. Infect. Dis.* **204**, S1066–S1074 (2011). 21987743 10.1093/infdis/jir348 PMC3203393

[74] Haddock, E. et al. A cynomolgus macaque model for Crimean-Congo haemorrhagic fever. *Nat. Microbiol.* **3**, 556–562 (2018). 29632370 10.1038/s41564-018-0141-7 PMC6717652

[75] Anhalt, H. & Marzi, A. Generation, recovery, and propagation of a recombinant vesicular stomatitis virus expressing the Marburg virus glycoprotein. *Methods Mol. Biol.* **2877**, 67–74 (2025). 39585614 10.1007/978-1-0716-4256-6_5

[76] Emanuel, J. et al. A VSV-based Zika virus vaccine protects mice from lethal challenge. *Sci. Rep.*10.1038/s41598-018-29401-x (2018). 10.1038/s41598-018-29401-x PMC6056530 30038228

[77] Tipih, T. et al. Favipiravir and ribavirin protect immunocompetent mice from lethal CCHFV infection. *Antivir. Res.* **218**, 105703 (2023). 37611878 10.1016/j.antiviral.2023.105703

[78] Reed, L. J. & Muench, H. A simple method of estimating fifty percent endpoints. *Am. J. Epidemiol.* **27**, 493–497 (1938).

[79] Hawman, D. W. et al. Immunocompetent mouse model for Crimean-Congo hemorrhagic fever virus. *Elife*10.7554/eLife.63906 (2021). 10.7554/eLife.63906 PMC7811403 33416494

