# Supplementary material for: Single dose VSV-based vaccine protects mice against lethal heterologous Crimean-Congo hemorrhagic fever virus challenge
Source: NPJ Vaccines. 2025 May 30;10:109. doi: 10.1038/s41541-025-01164-3 (PMC12125290; doi:10.1038/s41541-025-01164-3)
Supplement: Supplementary file 1 — nr-competing-interests [file 41541_2025_1164_MOESM1_ESM.pdf]

Disclosure of Potential Competing Interest

nature research

Journal Name:

npj Vaccines

Manuscript Number:

Manuscript Title:

Single dose VSV-based vaccine protects mice against lethal heterologous Crimean-Congo hemorrhagic fever virus challenge

Corresponding Author(s):

Heinz Feldmann & David Hawman

In the interests of transparency and to help readers form their own judgements of potential bias, *Nature Research* journals require authors to declare any competing financial and/or non-financial interests in relation to the work described in the submitted manuscript. The corresponding author is responsible for submitting a competing financial interests statement on behalf of all authors of the paper.

Financial competing interests

No, I declare the authors have no competing interests as defined by Nature Research, or other interests that might be perceived to influence the interpretation of the article. ☑

Yes, I declare the authors have competing interests as defined by Nature Research, or other interests that might be perceived to influence the interpretation of the article. ☐

If yes, please specify your competing interests in the box below, followed by the initials of the relevant author(s).

Funding
Research support to the author or their institution (including salaries, equipment, supplies and other expenses) by organizations that may gain or lose financially through this publication. A specific role for the funder in the conceptualization, design, data collection, analysis, decision to publish, or preparation of the manuscript, should be disclosed.

Employment
Recent (while engaged in the research project), present or anticipated employment by any organization that may gain or lose financially through this publication.

Personal financial interests
Stocks or shares in companies that may gain or lose financially through publication; consultation fees or other forms of remuneration (including reimbursement for attending symposia) from organizations that may gain or lose financially; patents or patent applications (awarded or pending) filed by the authors or their institutions whose value may be affected by publication. For patents and patent applications, disclosure of the following information is requested: patent applicant (whether author or institution), name of inventor(s), application number, status of application, specific aspect of manuscript covered in patent application.

Describe relevant competing interests below:

Non-financial competing interests

No, I declare the authors have no non-financial competing interests as defined by Nature Research, or other interests that might be perceived to influence the interpretation of the article. ☐

Yes, I declare the authors have competing interests as defined by Nature Research, or other interests that might be perceived to influence the interpretation of the article. ☑

If yes, please specify your competing interests in the box below, followed by the initials of the relevant author(s).

Non-financial competing interests can take different forms, including personal or professional relations with organizations and individuals. We would encourage authors and referees to declare any unpaid roles or relationships that might have a bearing on the publication process. Examples of non-financial competing interests include (but are not limited to):

- Unpaid membership in a government or non-governmental organization
- Unpaid membership in an advocacy or lobbying organization
- Unpaid advisory position in a commercial organization
- Writing or consulting for an educational company
- Acting as an expert witness

Describe relevant competing interests below:

Heinz Feldmann claims intellectual property of VSV-based filovirus vaccines. All other authors declare no competing interests.

I have read Nature Research's competing interest policy and declare that the above information is complete and correct.

Print Name: Heinz Feldmann

Signature: Heinz Feldmann Date: 1-6-2025

Springer Nature Limited, Registered Office: The Campus, 4 Crinan Street, London, N1 9XW, UK. Company number 785998.
