# Supplementary material for: Single dose VSV-based vaccine protects mice against lethal heterologous Crimean-Congo hemorrhagic fever virus challenge
Source: NPJ Vaccines. 2025 May 30;10:109. doi: 10.1038/s41541-025-01164-3 (PMC12125290; doi:10.1038/s41541-025-01164-3)
Supplement: Supplementary file 2 — Supplementary files [file 41541_2025_1164_MOESM2_ESM.pdf]

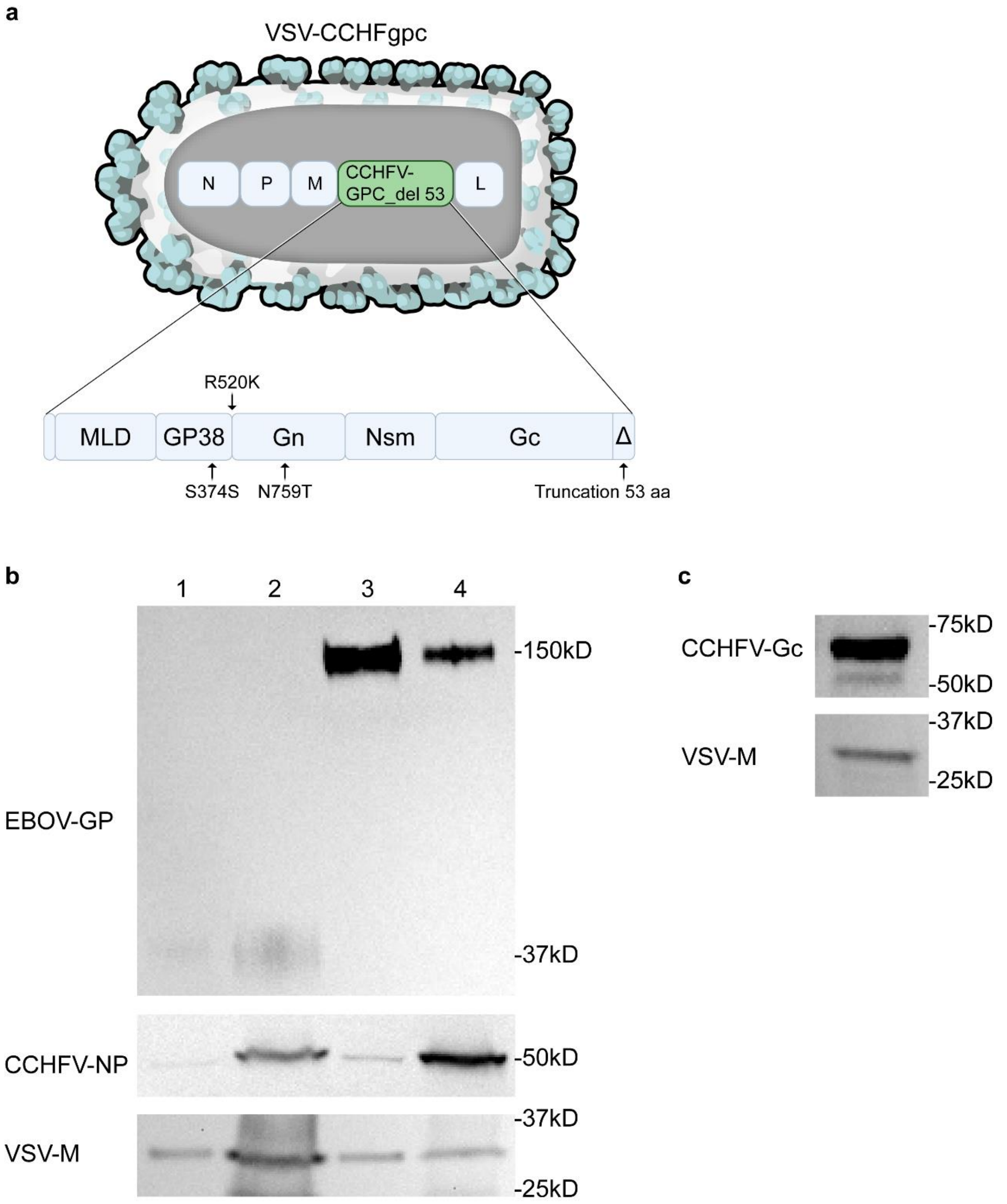

Supplementary Figure 1: Characterization of VSV vectors. a VSV-CCHFgpc vector design. The CCHFV-GPC was generated with a deletion of 53 amino acid residues off the carboxy terminal region of the Gc. Next generation sequencing revealed additional mutations, one synonymous (S374S) and two non-synonymous mutations (R520K & N759T) in the open reading frame of the CCHFV GPC. **b, c** Incorporation of antigens into VSV. Clarified supernatants and cell lysates of Vero E6 cells infected with different VSV vectors (MOI = 0.01) were analyzed for EBOV-GP, CCHFV-NP (lane 1: VSV-CCHFnp2 supernatant, VSV-CCHFnp2 cell lysate, VSV-CCHFnp1 supernatant, VSV-CCHFnp1 cell lysate) or CCHFV-Gc (**c**), from clarified VSV-CCHFgpc supernatant, incorporation by immunoblotting. VSV-M served a as control in (**b**) and (**c**). Graphical illustrations were prepared with Adobe Illustrator version 28.7 (public domain).

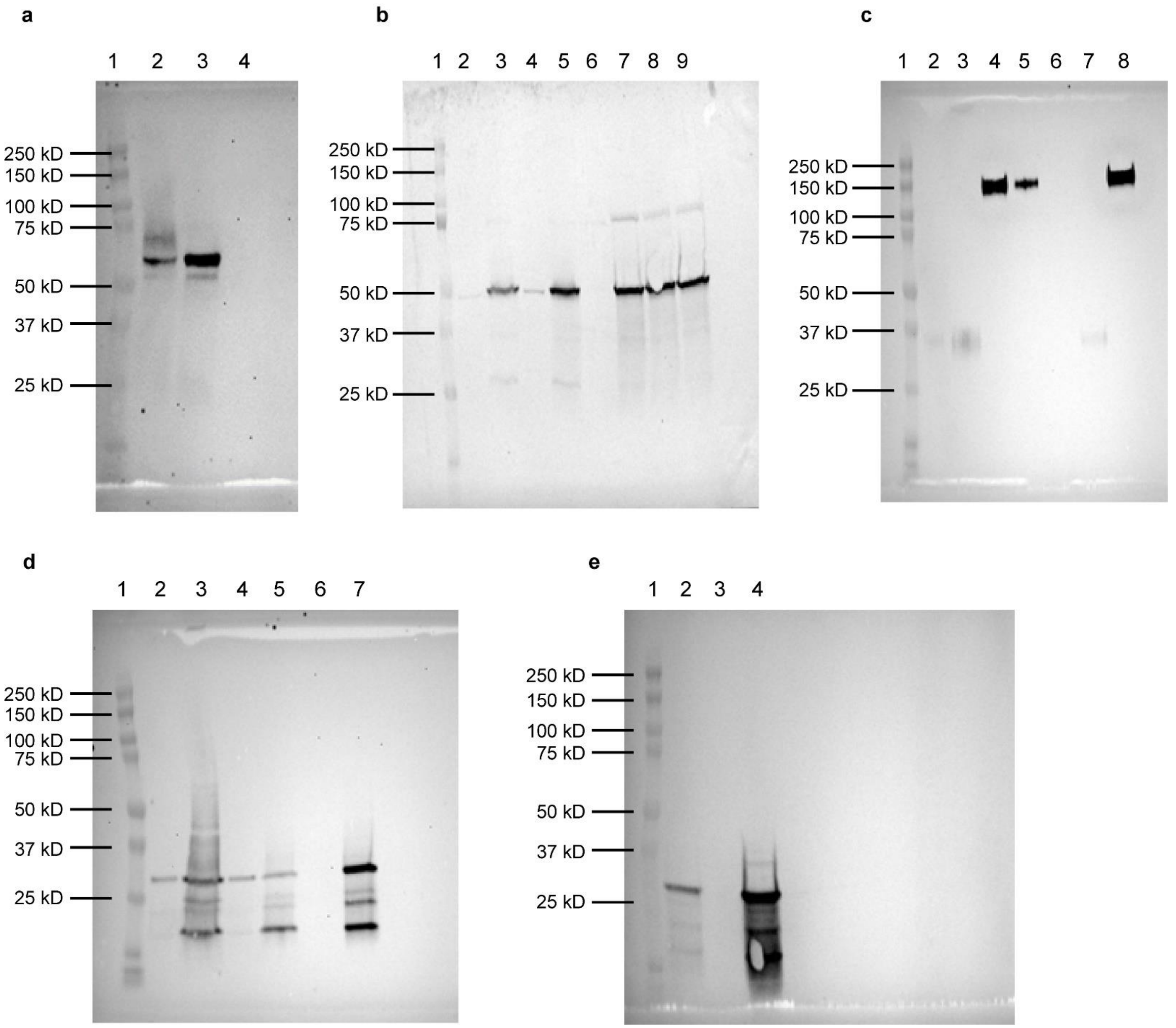

Supplementary figure 2. The full and uncropped images of Supplementary Figure 1b, c. a CCHFV-Gc detection using 11E7 monoclonal antibody. 1: Bio-Rad Precision Plus Protein Dual Color Standard, 2: positive control, 3: VSV-CCHFgpc, 4: negative control. **b** CCHFV-NP detection using CCHFV-NP specific sera raised in rabbits**.** 1: Bio-Rad Precision Plus Protein Dual Color Standard, 2: VSV-CCHFnp2 supernatant, 3: VSV-CCHFnp2 cell lysate, 4: VSV-CCHFnp1 supernatant, 5: VSV-CCHFnp1 cell lysate, 6: negative control, 7: positive control 1, 8: positive control 2, 9: positive control 3. **c** EBOV-GP detection using anti-EBOV-GP 12/1.1 monoclonal antibody: 1: Bio-Rad Precision Plus Protein Dual Color Standard, 2: VSV-CCHFnp2 supernatant, 3: VSV-CCHFnp2 cell lysate, 4: VSV-CCHFnp1 supernatant, 5: VSV-CCHFnp1 cell lysate, 6: negative control, 7: positive control 1 (EBOV ΔGC ΔMLD), 8: positive control 2 (EBOV-GP). **d** VSV-M detection using anti-VSV-M monoclonal antibody: 1: Bio-Rad Precision Plus Protein Dual Color Standard, 2: VSV-CCHFnp2 supernatant, 3: VSV-CCHFnp2 cell lysate, 4: VSV-CCHFnp1 supernatant, 5: VSV-CCHFnp1 cell lysate, 6: negative control, 7: positive control 1. **e** VSV-M detection using anti-VSV-M monoclonal antibody. 1: Bio-Rad Precision Plus Protein Dual Color Standard, 2: VSV-CCHFgpc, 3: negative control, 4: positive control. Graphical illustrations were prepared with Adobe Illustrator version 28.7 (public domain).

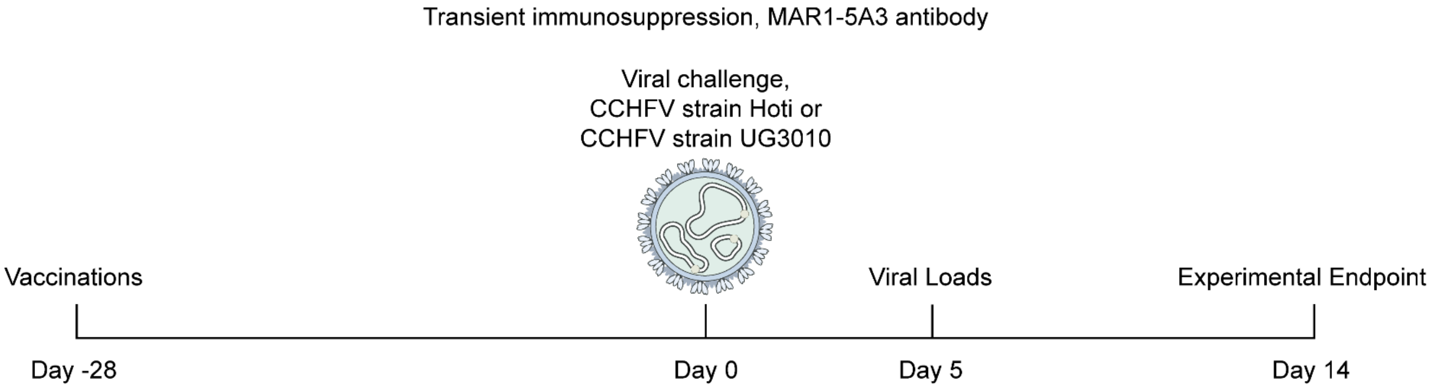

Supplementary Figure 3: Schematic representation of the study outline. Six-week-old C57BL/6J mice were vaccinated intraperitoneally (IP) with $1x10^4$ PFU VSV-CCHFnp1, VSV-CCHFnp2, VSV-CCHFgpc, or VSV-EBOV on day -28. On day 0 groups of 6 mice were euthanized and collected blood and spleen samples for evaluation of immune responses. Group of 14 animals were treated IP with MAR1-5A3 antibody and immediately challenged IP with 100 $TCID_{50}$ of either CCHFV strain Hoti or CCHFV strain UG3010. Groups of 6 animals were euthanized 5 days post-challenge and tissue samples and blood were collected for evaluation of viral load and histopathology. Groups of 8 animals were monitored for 14 days to evaluate protective efficacy of the vaccines. Surviving animals were euthanized on day 14 (study endpoint). Graphical illustrations were prepared with Adobe Illustrator version 28.7 (public domain).

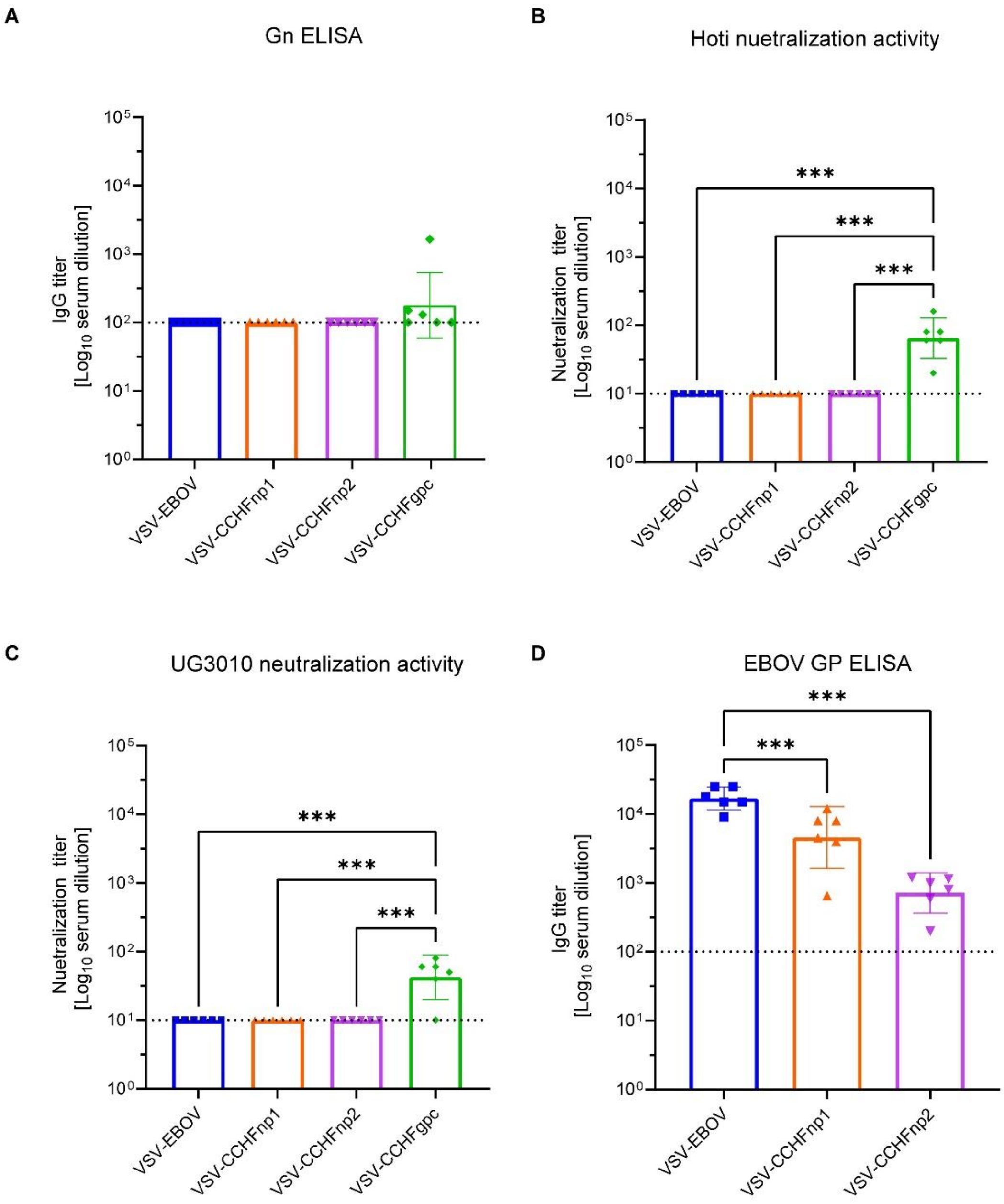

Supplementary Figure 4: Neutralizing antibody response. Six-week-old C57BL/6J mice were vaccinated intraperitoneally (IP) with 1x10^4 PFU VSV-CCHFgpc, VSV-CCHFnp1, VSV-CCHFnp2, or VSV-EBOV. 28 days post vaccination, animals were euthanized for analysis of immune responses. **a** IgG for CCHFV-Gn specific IgG antibodies using recombinant protein ELISA. CCHFV specific antibody neutralization titers against the CCHFV strain Hoti (**b)** or CCHFV strain UG3010 (**c**) was measured using a microneutralization assay. **d** EBOV-GP IgG specific antibodies was measured using recombinant protein ELISA. Dashed line represents ELISA cut-off for seropositivity (**a, d**) or neutraliation assay (**b, c**) limit of detection. The value was set at 3 standard deviations above the mean absorbance of wells that recived no serum for the former and logarithim base 10 of the intitial dilution of 1:10 for the latter. **a**, **b**, **c**, **d** Statistical significance was calculated using one-way ANOVA with Tukey's multiple comparison and results are indicated as *p < 0.05, ***p* < 0.01 and ****p* < 0.001. Comparisons with p values > 0.05 were not displayed. Data shown as geometric mean plus standard deviation.

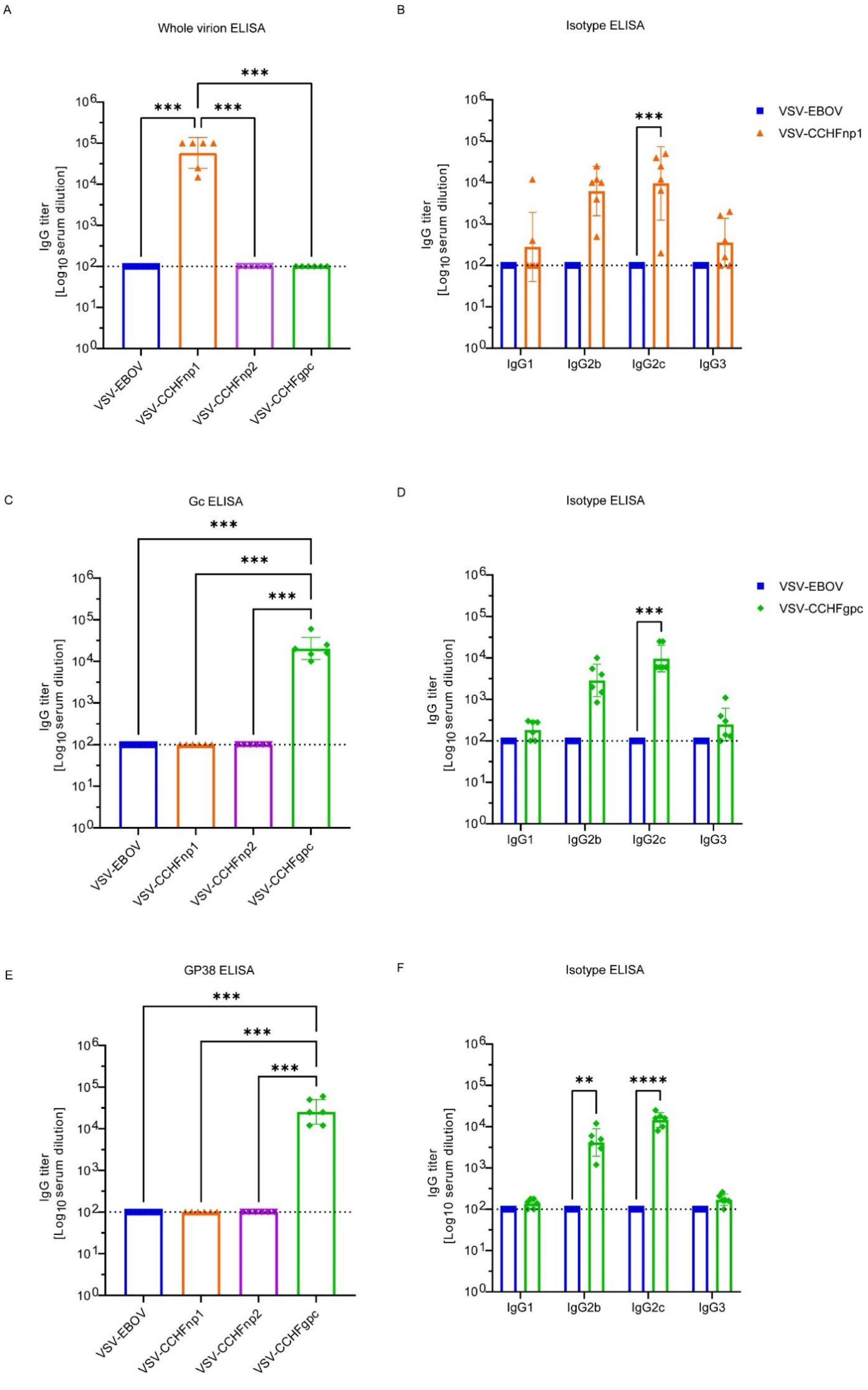
A
Whole virion ELISA
***
***
***
IgG titer
[Log10 serum dilution]
VSV-EBOV
VSV-CCHFnp1
VSV-CCHFnp2
VSV-CCHFgpc
B
Isotype ELISA
***
VSV-EBOV
VSV-CCHFnp1
IgG1
IgG2b
IgG2c
IgG3
C
Gc ELISA
***
***
***
D
Isotype ELISA
***
VSV-EBOV
VSV-CCHFgpc
E
GP38 ELISA
***
***
***
F
Isotype ELISA
**
****
IgG titer
[Log10 serum dilution]

Supplementary Figure 5: VSV-CCHFgpc and VSV-CCHFnp1 vaccination induce significant B cell responses. Six-week-old C57BL/6J mice were vaccinated intraperitoneally (IP) with $1x10^4$ PFU VSV-CCHFnp1, VSV-CCHFgpc, or VSV-EBOV on day -28. On day 0 groups of 6 mice were euthanized and blood and spleen samples were collected for evaluation of immune responses. Whole virion ELISA (**a**) or specific isotypes (**b**) was used to detect IgG responses elicited by the CCHFV-NP vaccines. CCHFV-Gc specific IgG antibodies were evaluated by recombinant protein ELISA (**c**) or specific isotypes (**d**). CCHFV-GP38 specific IgG antibodies were evaluated by recombinant protein ELISA (**e**) or specific isotypes (**f**). Dashed line represents cut-off for seropositivity which was set at 3 standard deviations above the mean absorbance of wells that recived no serum. Statistical significance was calculated using one-way ANOVA with Tukey's multiple comparison (**a**, **c**, **e**) or two-way ANOVA with Sidak's multiple comparison (**b**, **d**, **f**) and results are indicated as $*p < 0.05$, $**p < 0.01$ and $***p < 0.001$. Comparisons with p values > 0.05 were not displayed. Data shown as geometric mean plus standard deviation.

Supplementary Table 1: Histopathology matrix in heterologous challenged animals

	VSV-EBOV					VSV-CCHFnp1					
Liver											nsf
inflammation, degeneration, necrosis	4	4	4	4	4	2	3	3	3	2	
background subacute sinusoidal inflammation/coag necrosis											
CCHF IHC	5	5	5	5	5	1	3	2	2	1	0
Spleen						nsf				nsf	nsf
red pulp necrotic debris	2	2	2	3 w/fibrin	2		2	1	1		
white pulp necrosis/apoptosis	4	4	4	4	4		1	0	0		
red pulp macrophage proliferation	y	y	y	y	y	n	y	y	n	n	n
CCHF IHC red pulp predominantly	4	4	4	5	4	1barely	3	1	1	1barely	0

	VSV-CCHFnp2						VSV-CCHFgpc					
Liver		nsf										
inflammation, degeneration, necrosis	4		4	4	3	3	3	3	2	4	2	3
background subacute sinusoidal inflammation/coag necrosis												
CCHF IHC	4	0	5	5	3	3	3	3	1	4	1	2
Spleen		nsf							nsf		nsf	
red pulp necrotic debris	2		2	2	1	2	y & splenitis	y & splenitis	n	y	n	y & splenitis
white pulp necrosis/apoptosis	0		3	3	0	2	y	y	n	y	n	n
red pulp macrophage proliferation	y	n	y	y	n	y	y	y	n	y	n	y
CCHF IHC red pulp predominantly	3	0	4	5	2	5	3	3	1barely	3	1	1

nsf = no significant findings
y = yes
n = no

Percent affected and distribution
0 = none
1 = 1-10% (focal)
2 = 11-25% (multifocal)
3 = 26-50% (multifocal to coalescing)
4 = 51-75% (multifocal to calescing)
5 = 76-100% (diffuse)

EMH = extramedullary hematopoiesis
IHC-immunohistochemistry

Table S2: Histopathology matrix in momologous challenged animals

	VSV-EBOV						VSV-CCHFnp1						VSV-CCHFgpc					
Liver																		
inflammation, degeneration, necrosis	3	3	3	3	3	3	3	3	3	3	2	3	0	3	2	3	3	1
background subacute sinusoidal inflammation/coag necrosis																		
CCHF IHC	5	5	5	5	5	5	4	3	3	1	1	4	0	2	1	1	3	1 barely
Spleen	nsf	nsf	nsf	nsf	nsf	nsf	nsf	nsf	nsf	nsf	nsf	nsf	nsf	nsf	nsf	nsf	nsf	nsf
red pulp necrotic debris																		
white pulp necrosis/apoptosis																		
red pulp macrophage proliferation	y	y	y	y	y	y	y					y					y	
other	EMH	EMH	EMH		EMH		EMH					EMH			EMH			EMH
CCHF IHC red pulp predominantly	4	4	4	4	4	4	2	2	1	1	1 barely	3	0	1	1	1	3	0

nsf = no significant findings
y = yes
n = no

Percent affected and distribution
0 = none
1 = 1-10% (focal)
2 = 11-25% (multifocal)
3 = 26-50% (multifocal to coalescing)
4 = 51-75% (multifocal to calescing)
5 = 76-100% (diffuse)

EMH = extramedullary hematopoiesis
IHC-immunohistochemistry
